# Towards molecular controlled magnonics†

**DOI:** 10.1039/d4na00230j

**Published:** 2024-05-01

**Authors:** Alberto M. Ruiz, Gonzalo Rivero-Carracedo, Andrey Rybakov, Sourav Dey, José J. Baldoví

**Affiliations:** a Instituto de Ciencia Molecular, Universitat de València 46980 Paterna Spain j.jaime.baldovi@uv.es

## Abstract

Magnonics is an emerging field broadly recognized as a paradigm shift for information technologies based on the use of spin waves. However, the low flexibility and variety of the existing systems still hamper their applications. Herein, we propose an unprecedented chemical approach to magnonics based on the creation of hybrid molecular/2D heterostructures. We analyse the modulation of the magnetic properties, magnon dispersion and spin dynamics of a single layer of CrSBr after the deposition of sublimable organic molecules *via* first-principles calculations. Our results predict a modulation of magnetic exchange, a shift in the magnon frequencies and an enhancement of their group velocities up to ∼7%. Interestingly, we find a linear correlation between these effects and the donor character of the molecules. This will pave the way for the design of a new class of magnonic materials that can be selectively tailored by a chemical approach.

## Abstract 2

This work provides an unprecedented approach to magnonics based on the creation of molecular/2D heterostructures using organic molecules and single-layer CrSBr.

## Graphical abstract

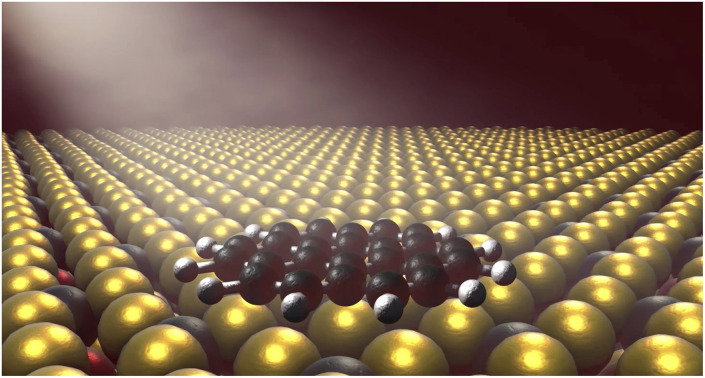

## Introduction

Since the discovery of graphene,^1^ two-dimensional (2D) materials have attracted extensive attention from the scientific community. Their low dimensionality, flexibility and tunability make them ideal candidates in the search for advanced quantum materials.^2–4^ In this context, the recent emergence of van der Waals (vdW) magnetic materials that retain long-range magnetic order down to the 2D limit, such as the layered antiferromagnets CrI_3_ or CrSBr,^5,6^ provides unprecedented opportunities for spintronics and magnonics.^7^ In this class of materials, the magnetic properties can be tuned by electric fields,^8^ electrostatic doping,^9^ strain engineering^10^ or light^11^ and can hold long-lived spin waves (SWs) in the GHz to THz range,^12,13^ thus opening new horizons for information and quantum technologies. Magnonics is a rapidly growing research field that investigates the transmission, storage, and processing of information by using SWs,^14^ whose quanta are called magnons. It is based on the propagation of collective magnetic excitations as an alternative to the motion of electric charges, leading to nano-sized devices with lower power consumption, a tunable spectrum and shorter wavelengths than conventional electronic ones.^15,16^ The recent detection of magnons in 2D magnets offers several advantages. These materials represent the limit of miniaturization, can be tuned through vdW stacking, and are integrable with silicon technology.^17,18^

A promising and unexplored strategy to control the magnetic properties and, therefore, the spin excitation spectra of a 2D magnet is to design hybrid molecular/2D heterostructures using a chemical approach. The versatility of chemistry supplies an almost infinite assortment of possibilities, pushing this disruptive technological approach beyond its state-of-the-art. Furthermore, molecules can self-assemble *via* supramolecular interactions forming crystalline 2D architectures on the surface of inorganic materials. The potential of this chemical approach has already been demonstrated in modern electronics, enhancing the efficiency of several devices such as OLEDs, spin-valves and sensors, among others,^19,20^ and tuning the properties of 2D materials.^21–24^ Moreover, the deposition of organic molecules has been shown to be an efficient technique to manipulate the local magnetic moments of magnetic substrates,^25^ which can be exploited to selectively control magnon transport in these systems, as has already been theoretically suggested using gas species.^26^

In this work, we provide a new approach to magnonics based on the design of hybrid molecular/2D heterostructures. As a model system, we select the 2D semiconducting ferromagnet CrSBr, whose magnons have been experimentally detected and exhibits the highest Curie temperature (*T*_C_) among 2D magnets.^6,27–31^ As molecular components, we focus on sublimable and planar organic molecules with π(pz) electrons,^32^ which have been proven to display a noticeable interaction with different substrates.^33–35^ In particular, we investigate electron donors such as perylene, coronene and tetrathiafulvalene (TTF), as well as tetracyanoquinodimethane (TCNQ), which is an electron acceptor, exploring the effect of molecular adsorption on the electronic structure, magnon dispersion and spin dynamics of CrSBr. Our results lay the first stepping stone for the development of a next-generation of chemically controlled nanomaterials for magnonic applications.

## Results and discussion

**Fig. 1:**
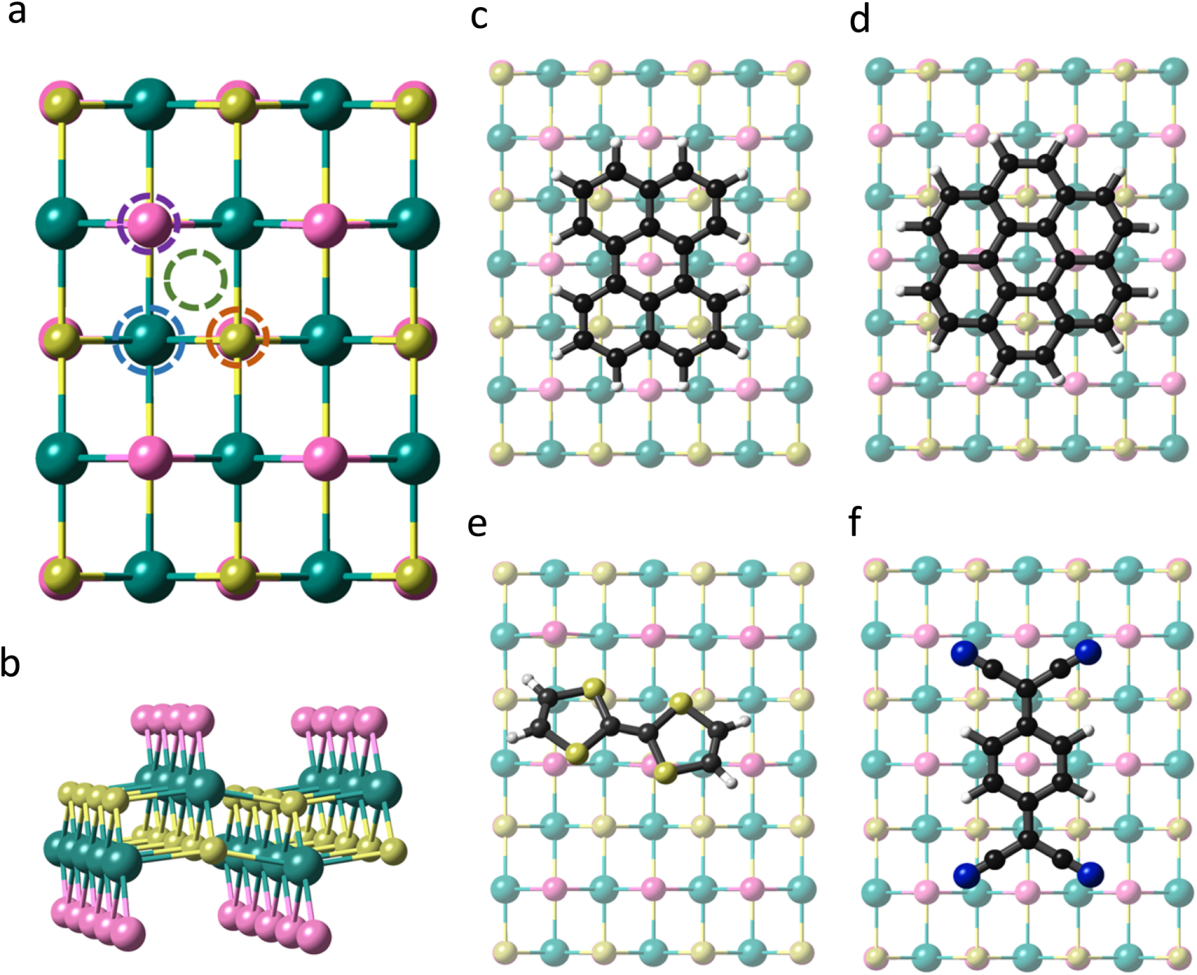
(a) Top view of CrSBr monolayer indicating the studied adsorption sites, namely Cr-top (blue circle), S-top (orange circle), Br-top (purple circle) and hollow-top (green circle). (b) Lateral view of CrSBr monolayer. (c–f) Top views of the optimized adsorption geometries of perylene, coronene, TTF and TCNQ, respectively, on a single layer of CrSBr.

CrSBr is a layered magnetic material that crystallizes with an orthorhombic structure in the space group *Pmmm*. Within each layer the Cr atoms are arranged in a distorted octahedral environment, where the d orbitals are split into two sets of energy levels, namely, t_2g_ (d_*xy*_, d_*xz*_, and d_*yz*_) and e_g_ (d_*z*^2^_ and d_*x*^2^−*y*^2^_). Each Cr atom is linked to its nearest Cr neighbour through S and Br along *a* and just by S along the *b* and diagonal directions (Fig. 1). According to our first-principles calculations, the optimized lattice parameters of the monolayer are *a* = 3.601 Å and *b* = 4.818 Å, in good agreement with previous studies.^36,37^

Firstly, we investigate the effects of molecular adsorption on the properties of a single layer of CrSBr by designing hybrid molecular/2D heterostructures based on perylene, coronene, TTF and TCNQ, which are placed at different adsorption sites (Fig. 1a). For each molecule, we optimize the four configurations considering their relative orientation with respect to the substrate and investigate the interaction strength between the molecules and the 2D material by determining the adsorption energy (*E*_ads_) (See Table S1 and Fig. S1†).

The optimized structures are presented in Fig. 1c–f. The most stable heterostructure is TTF/CrSBr with an *E*_ads_ = −1.55 eV, followed by perylene, coronene and TCNQ with values of −1.35, −1.18 and −0.65 eV, respectively. Note that for all the molecules and adsorption sites the molecular deposition is favourable, indicating that their physisorption is an exothermic process and thermodynamically stable. In this regard, these molecules have already been predicted and confirmed experimentally to be stable over other substrates such as Au(111) or graphene, based on similar values of *E*_ads_.^38–40^

As TTF/CrSBr is the most energetically favourable heterostructure, we study the possibility of enhancing such interaction by (i) replacing the S atoms of TTF by Se and (ii) introducing outer methyl groups, *i.e.* changing electronegativity and the donor character of the molecule (Fig. S2†). The main effect is provided by the latter, increasing the adsorption energy up to −2.23 eV (Table S2†). This can be attributed to the donor character of the added substituents, which allows a more efficient charge transfer to the substrate. On the other hand, the replacement of S by Se does not trigger any significant modification. Hence, we consider TTF-CH_3_ in the subsequent calculations.

**Fig. 2:**
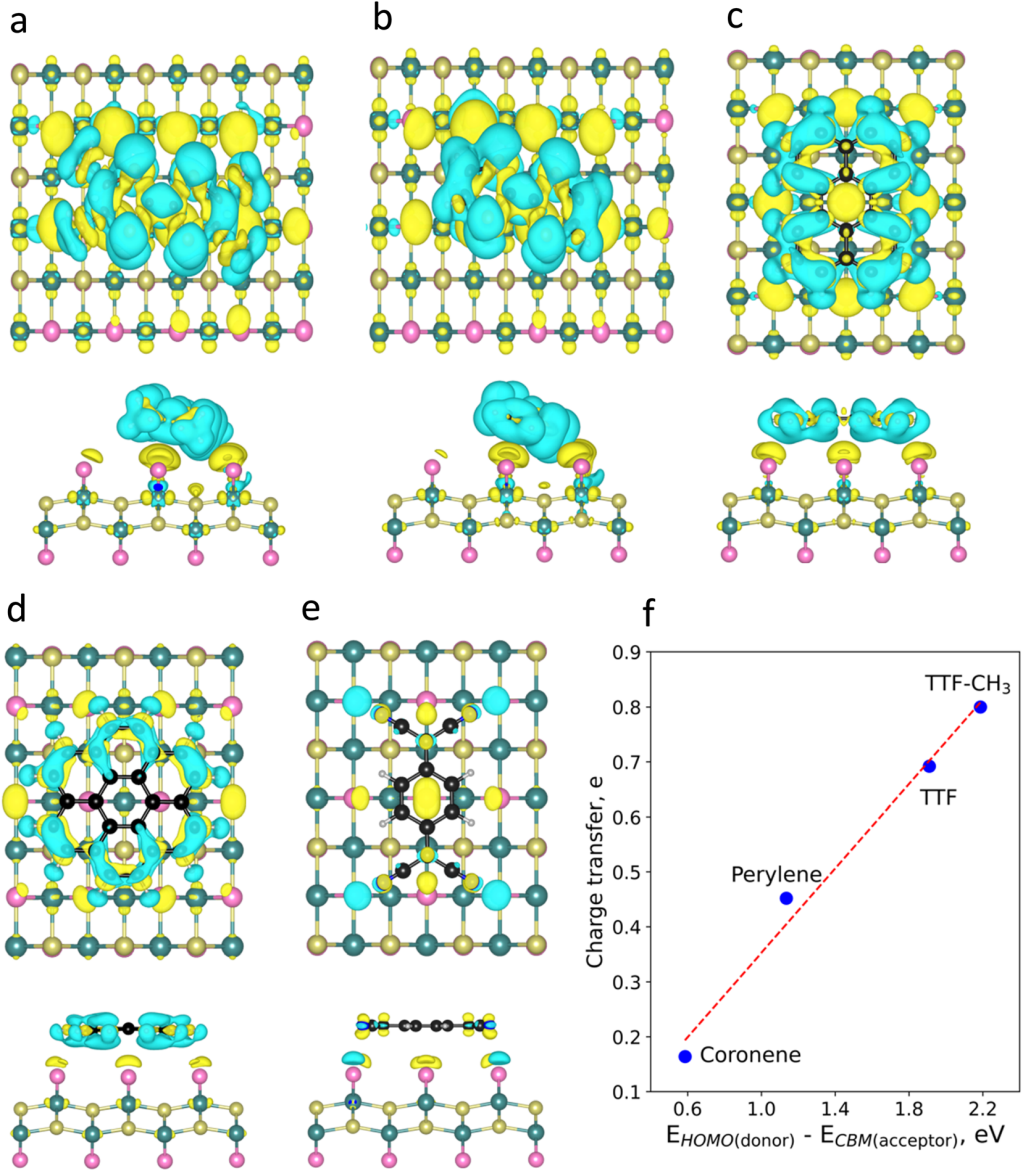
Charge density difference (CDD) plots of (a) TTF-CH_3_, (b) TTF, (c) perylene, (d) coronene, and (e) TCNQ. Blue and yellow colours represent regions with charge depletion and accumulation, respectively. (f) Evolution of charge transfer with the energy difference between the HOMO of the donor molecules and CBM of CrSBr.

To visualize the charge redistribution in the heterostructures, we represent the charge density difference (CDD) (Fig. 2). For the donor molecules, the charge depletion mainly occurs in the aromatic rings and the charge accumulation in the neighbouring Br atoms of the substrate due to their high electronegativity. Furthermore, we observe that TTF-CH_3_ and TTF are tilted with respect to the surface (around 24°), while the rest of the systems remain planar. This is attributed to the favourable overlap between the highest occupied molecular orbital (HOMO) and the π orbitals of the close Br atoms from the substrate, thus intensifying the binding strength (see Fig. S3†). By contrast, in the case of TCNQ, there is a negligible electron density accumulated in the cyano groups due to the acceptor character of the molecule. We also plot the spin density, showing that in all cases the molecules get spin polarized (Fig. S4†).

Then, we perform a Bader charge transfer analysis for the five molecules in their most stable configurations to quantify the electron density flow in the interface (Table S3†). It is shown that the donor-like molecules supply electron density to the substrate. In particular, TTF-CH_3_ exhibits the highest charge transfer (0.80*e*), followed by TTF, that donates 0.69*e*. Additionally, perylene and coronene give 0.45*e* and 0.16*e*, respectively. On the other hand, TCNQ accepts a negligible number of electrons (0.03*e*) from CrSBr. One can observe a linear relation between the charge transfer of the donor molecules and the energy difference of the HOMO and the conduction band minimum (CBM) of CrSBr (Fig. 2f). This proves that such energy difference plays a predominant role in the interaction between the molecules and the substrate, where a high difference between donor's HOMO and acceptor's CBM energy levels gives rise to a substantial charge transfer.

**Fig. 3:**
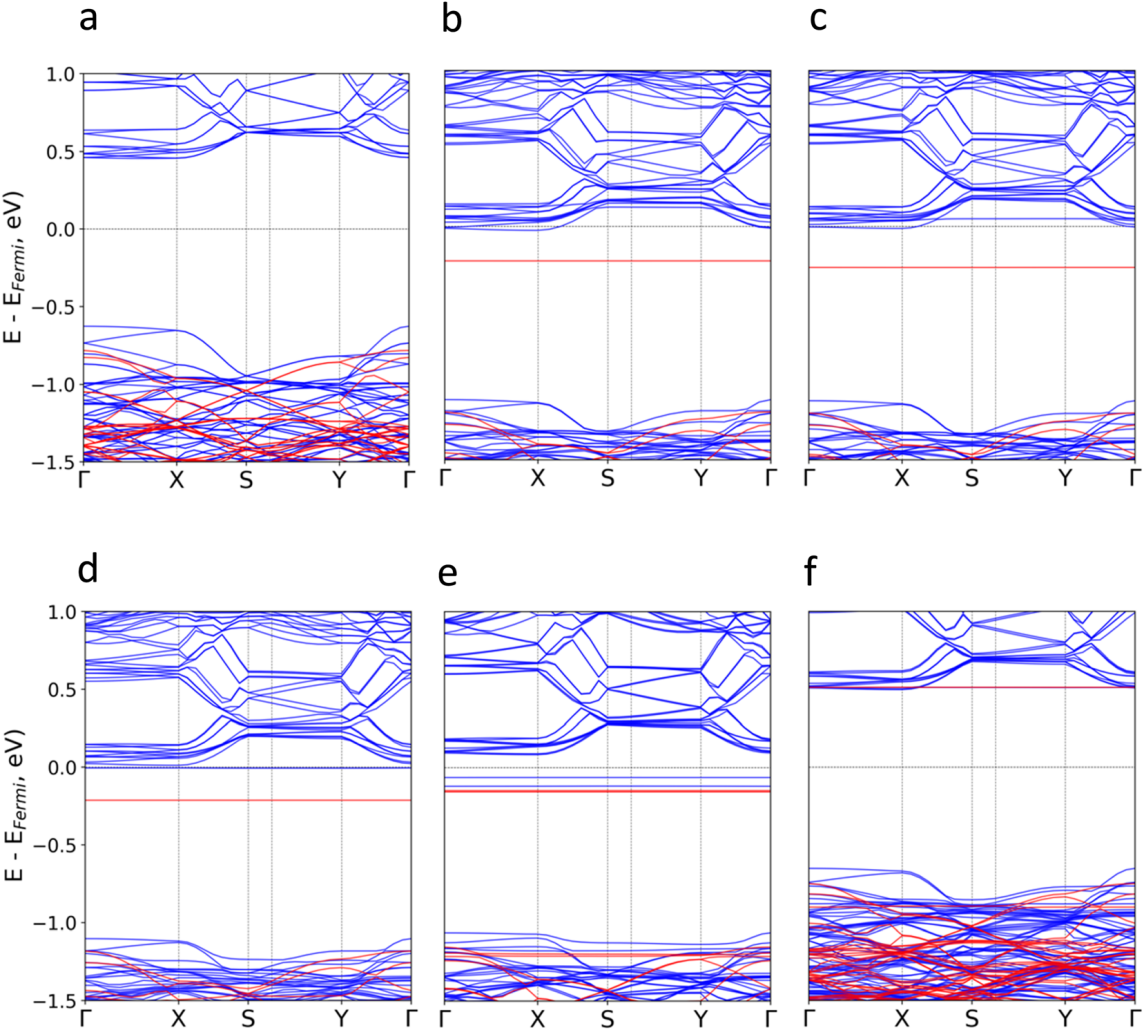
Calculated electronic band structure for (a) pristine CrSBr and the hybrid molecular/2D heterostructures based on (b) TTF-CH_3_, (c) TTF, (d) perylene, (e) coronene, (f) TCNQ. Blue (red) colour represents spin up (down) energy bands.

Subsequently, we investigate the effect of the molecular adsorption on the electronic band structure and density of states (Fig. 3 and S5–S16†). According to our calculations, the intrinsic band gap of the material (1.09 eV) is not modified, but new localized states around the Fermi level (*E*_f_) for TTF-CH_3_, TTF, perylene and coronene are introduced. Their donor nature pushes upwards the *E*_f_, and thus empty states from the conduction bands of CrSBr are populated. The poor hybridization between TCNQ and CrSBr results in the absence of molecular states around the *E*_f_. For all systems, the discrete levels of the molecules are not broadened, but they keep rather localized (represented as flat bands near the *E*_f_). This is compatible with a physisorption process, where the interfacial interactions are mainly governed by vdW forces.

**Fig. 4:**
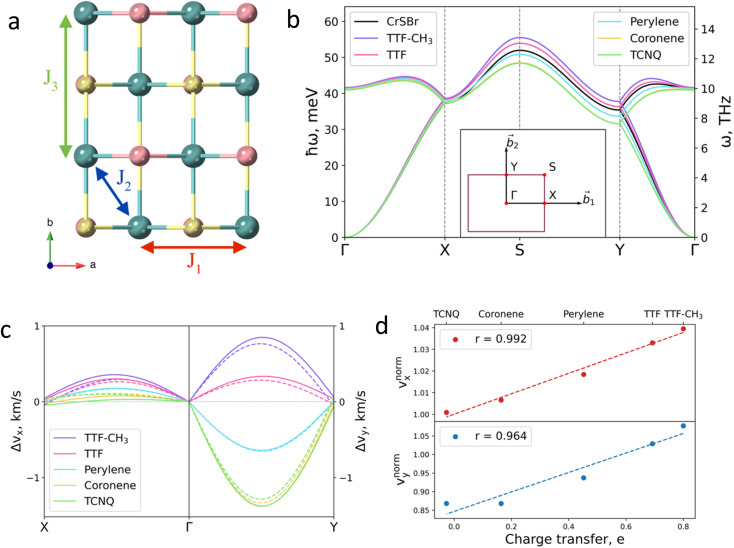
(a) Top view of CrSBr monolayer. *J*_1_, *J*_2_ and *J*_3_ represent the magnetic exchange parameters between first, second and third neighbours, respectively. (b) Effect of the deposited molecules on magnon dispersion. Insert shows the *k*-path. (c) Changes in group velocity *v*_*x*_, along *Γ*–*X* path and in *v*_*y*_ along *Γ*–*Y* path for each molecule. Values are given with respect to the pristine material. Solid (dashed) lines correspond to the acoustic (optical) branch. (d) Relation between the changes in the group velocity due to the molecules and the charge transfer imposed by each molecule. Changes in group velocities are normalized with respect to the TTF-CH3. Insert shows the Pearson's r (see ESI for details†).

Next, we investigate the effect of the deposition of the organic molecules on the magnetic exchange interactions. Note that in single layer CrSBr, the magnetic exchange constants can be described considering superexchange mechanisms between neighbouring Cr atoms mediated by the p orbitals of Br and S up to third nearest-neighbours, thus resulting in *J*_1_, *J*_2_ and *J*_3_ (Fig. 4a). First, *J*_1_ is defined as the interaction between two Cr atoms along the *a* axis, mediated by S (*α* = 95.8°) and Br (*β* = 89.8°). *J*_2_ accounts for the interaction between Cr atoms along the *ab* diagonal direction and mediated by two S atoms (*γ* = 97.5°) while *J*_3_ is defined as the interaction between Cr–S–Cr (*δ* = 157.6°) along the *b* crystallographic direction. According to the Goodenough–Kanamori rules,^41^ ferromagnetic (FM) superexchange interactions are favoured between the Cr^3+^ ions with partially filled t_2g_ orbitals and bond angle close to 90° (*α*, *β* and *γ*), whereas antiferromagnetic (AFM) coupling is expected for a bond angle of 180°. This results in FM coupling along the *a* axis and *ab* diagonal direction, governed by the t_2g_–e_g_ pathway, and a competition between FM and AFM coupling along *b*.

Magnetic exchange interactions are extracted by mapping total energies from different magnetic configurations (Fig. S17†) on a classical Heisenberg Hamiltonian. The isotropic magnetic exchange parameters for CrSBr monolayer are *J*_1_ = 3.11 meV, *J*_2_ = 3.87 meV, and *J*_3_ = 2.76 meV, which are in good agreement with neutron diffraction experiments and previous theoretical results.^27,42^ Upon molecular deposition, we observe that the FM ground state is preserved for all five molecules (Table S5†). As a general trend, *J*_1_ and *J*_2_ are almost insensitive, whereas *J*_3_ is enhanced for TTF-CH_3_ and TTF, decreasing in the case of perylene, coronene and TCNQ (Table S5†). The modification of the magnetic exchange comes from the competition between two different phenomena, namely (i) the molecular induced distortion of the substrate and (ii) the interaction effects between the 2D system and the molecules.

To account for the geometrical-like contribution we obtain the magnetic exchange parameters only by considering the distorted substrate in our calculations (Table S6†). For the five molecular-induced distorted geometries, *J*_1_ and *J*_2_ are slightly enhanced and reduced, respectively, with respect to the clean surface. On the other hand, *J*_3_ decreases drastically, reaching a minimum value of 1.90 meV (−31.16%) for the TTF-CH_3_/CrSBr heterostructure. These results agree well with the small changes of the angles mediating *J*_1_ and *J*_2_ and the substantial modification of the one along *J*_3_ (Table S7†). Our findings reveal that the latter (*δ*) is enhanced for the five systems, with a maximum change of +5.1° for TTF-CH_3_ with respect to pristine CrSBr. This larger value results in a better alignment of the d_*z*^2^_–p_*z*_–d_*z*^2^_ orbitals of Cr and S atoms, leading to an enhancement of the AFM channel.^43^

When considering the interaction effects between the molecules and CrSBr, all three FM exchange interactions are increased for TTF-CH_3_, TTF, perylene and coronene with respect to the distorted geometries (Table S5†). This is due to their electron-donor character. The electrons transferred to CrSBr occupy the empty e_g_ orbitals of Cr, (Fig. S19†) which could generate the Hund's coupling exchanges with other local spins of Cr. Such effect has been previously reported to enhance the magnetic coupling due to the emergence of a double exchange between neighbouring Cr atoms.^44^ This is also consistent with the change in magnetic moments of Cr atoms, which are higher in the region covered by the molecules (Fig. S21†). Note that as TCNQ receives a negligible amount of charge from the substrate, the change in magnetic coupling is solely governed by the structural molecular-induced distortion. To shed light into the origin of the enhancement of ferromagnetism when considering the molecules, we apply electron doping (selecting the same number of electrons as the ones transferred in the hybrid heterostructures) to the distorted CrSBr structures without considering the adsorbed molecules in the calculations. Then, we analyse the strength of magnetic couplings (Table S8 and Fig. S22, S23†), concluding that the FM interactions are enhanced in the same magnitude than upon molecular deposition for the four systems (with a maximum error of 9.8%). Hence, charge transfer is the main source of interaction between the molecules and the 2D material, allowing us to correlate it with the modulation of magnetic exchange. We also test the effects of an increased molecular coverage (from a molecular density of 1.6 × 10^13^ cm^−2^ to 7.2 × 10^13^ cm^−2^) to simulate a more realistic monolayer of TTF molecules deposited on CrSBr. This results in an enhancement of *J*_1_, *J*_2_ and *J*_3_ (Table S9†) due to higher electron density being transferred. We also quantify the impact of the deposition of the molecules on the *T*_C_ of the substrate (Fig. S24†). For the pristine system, our calculations reveal a value of 140 K, which aligns well with experimental observations (146 K).^6^ We observe that *T*_C_ increases with the donor character of the molecules, where TTF-high coverage results in a remarkable enhancement of *T*_C_ of 35 K, followed by TTF-CH_3_ and TTF showing enhanced values of ∼8 K. On the other hand, the *T*_C_ of perylene remains almost unchanged, whereas for coronene and TCNQ there is a reduction of 3 and 10 K, respectively.

To demonstrate the effect of the chemical tuning on the magnetic excitations, we calculate the magnon dispersion relation using the Holstein–Primakoff^45^ transformation (Fig. 4b) for the pristine and the hybrid molecular/2D heterostructures. The simulation of the magnon dispersion of the pristine system shows a good agreement with experimental bulk spin wave spectrum^27^ (Fig. S25†). According to our calculations, the main effects of the molecular adsorption can be observed in high energy magnons. This effect could be detected by a frequency shift in inelastic neutron scattering measurements and will have an impact in the amplitude of electrically or thermally-excited magnon currents in the material. As previously described, molecular deposition affects the substrate *via* two main mechanisms: structural distortion and charge transfer. The distortion of the substrate influences the magnon dispersion in a similar manner for all considered molecules, resulting in the decrease of magnon energy for the whole *k*-path but *Γ*–*X*, where the magnon energies remain effectively constant, following the behavior of *J*_1_ (see Fig. S26†). On the contrary, the effect of charge transfer mechanism on the magnons is more selective and follows a direct relation with the donor character of the molecules (see Fig. S27†). In addition, we observe that a higher coverage of TTF results on a more pronounced effect on the magnon dispersion arising from its higher electron transfer to the substrate (Fig. S28†).

The molecular effects on the spin waves are further illustrated by investigating the group velocities of magnon propagation, which can be phenomenologically described for the two orthogonal directions due to the symmetry of the material: *v*_*x*_ (along *Γ*–*X*) and *v*_*y*_ (along *Γ*–*Y*). The changes of the group velocities caused by the molecules are presented in Fig. 4c (see Fig. S29 and S30† for the absolute values). The major change is observed in *v*_*y*_ (in the range of −13% to +7% for TCNQ and TTF-CH_3_, respectively), whose behavior is mainly governed by the evolution of *J*_3_. The evolution of *v*_*x*_ and *v*_*y*_ with respect to the distorted substrates is reported in Fig. S31.† It confirms that the charge transfer is the main mechanism that governs the evolution of group velocities, given that the tendency is almost identical for both calculations, *i.e.* considering the molecules and just the electron doping effect.

Finally, we rationalize the full effect of the molecules by plotting the relative group velocities normalized with respect to the values of pristine CrSBr (Fig. 4d; see supporting section 4.2 and Fig. S32† for details). As one can observe, the evolution of relative group velocities with respect to the charge transfer follows a linear trend for the low-energy magnons (acoustic branch). Note that this dependency accounts for both mechanisms: structural distortion and charge transfer, which allows the rational design of hybrid molecular/2D heterostructures with enhanced spin wave group velocities.

In this study we select widely known sublimable donor and acceptor organic molecules, whose chemical complexity is relatively small. We find that chemical modifications to TTF such as methylation can enhance the hybridization by increasing the electron-donating behaviour. Therefore, this versatility could be exploited in the search of new molecular species that are suitable for improving the magnetic properties and SWs transport. In this context, highly donor species such as ferrocene, pentacene, fullerene or phthalocyanine derivatives could be potential interesting candidates for this purpose.

Furthermore, we envision similar tendencies for the relation between HOMO–CBM and charge transfer for CrI_3_ and Cr_2_Ge_2_Te_6_, where both present a d^3^ configuration for Cr. In this regard, electron donor molecules would favour the enhancement of FM interactions. Also, we expect that the FM exchange could be enhanced upon deposition of electron acceptor molecules, due to the lower electronegativity of I and Te with respect to Br in CrSBr, which would result in the opening of a new t_2g_–t_2g_ FM channel. In that sense, F_4_-TCNQ has been proven to have a strong acceptor character due to the high electronegativity of the fluorine groups.^46^

The charge transfer effect on the magnetic couplings has also been studied in the transition metal phosphorus trisulfides (MPS_3_) family. In this regard, FM interactions have been enhanced on MnPS_3_ (d^5^) upon electrostatic hole doping due to the opening of a new e_g_–e_g_ FM channel.^47^ In addition, an AFM-ferrimagnetic transition (and *vice versa*) has been observed in NiPS_3_ (d^8^) for different levels of electron doping.^48^ For this reason, we anticipate a similar behaviour for electron acceptor (donor) molecules adsorbed on MnPS_3_ (NiPS_3_).

Our results regarding chemical tuning of magnons might be extrapolated to bulk magnetic materials. In this context, Yttrium Iron Garnet (YIG) is leading the research interest in magnonics due to its very high operative temperature and low damping effects, being extensively experimentally studied. Specifically, for Ce-doped YIG thin films, a reduction of the AF magnetic coupling between Fe atoms in two different coordination environments has been attributed to charge transfer.^49^ This is originated from the partial reduction of Fe^3+^ to Fe^2+^, causing a depletion of the overall magnetic moment. For such reason, the use of electron donor molecules, which show a strong hybridization, can be exploited in the search of advanced systems displaying a similar behaviour.

While the adsorption of an organic molecule has proven to be an effective strategy to manipulate the local magnetic moments of the magnetic substrate, the latter can induce spin-polarized hybridized molecular states around the Fermi energy. Crucially, external stimuli such as light or electric voltages could be used to reach these excitations, allowing an active control of the spinterface. In that sense, a shift in the magnetic dipole at the spinterface might cause a disturbance of the local magnetic order of the magnetic material, driving the system out of equilibrium and generating a SW.

## Conclusions

In summary, we have investigated the effect of depositing sublimable organic molecules on the magnetic properties and magnon dynamics of the layered 2D magnet CrSBr by means of first-principles calculations. The main finding of this work is the demonstration of a molecular-driven selective modification of the magnon propagation speed within a maximum range of ∼20%. Importantly, we prove that the charge transfer at the interfaces is the main mechanism governing the modulation of the spin waves. This has relevant implications for future rational design because we demonstrate that (i) the energy difference between HOMO of the molecules and conduction band minimum of the material, as well as (ii) the relative group velocities, follow a linear relation with the charge transfer between molecule and substrate. This will allow to provide a high-throughput screening of molecular species and magnetic materials in order to select the most suitable hybrid heterostructures. Our results pave the way to the development of new materials for molecular controlled magnonics.

## Methods

We carried out spin polarized density-functional theory (DFT) calculations on single-layer CrSBr (the unit cell containing 2 Cr, 2 S and 2 Br atoms) using the Quantum ESPRESSO package.^50^ The generalized gradient approximation (GGA) was used as the exchange-correlation functional in the formalism of Perdew–Burke–Ernzerhof (PBE).^51^ To consider the electron correlation of 3d orbitals of Cr, we selected an effective Hubbard *U*_eff_ = 3 eV in the simplified version of Dudarev *et al.*^52^ The atomic coordinates and lattice parameters were fully optimized using the Broyden–Fletcher–Goldfarb–Shanno (BFGS) algorithm^53^ until the forces on each atom were smaller than 1 × 10^−3^ Ry au^−1^ and the energy difference between two consecutive relaxation steps was less than 1 × 10^−4^ Ry. The electronic wave functions were expanded with well-converged kinetic energy cut-offs for the wave functions (charge density) of 60 (600) Ry. We used standard solid-state ultrasoft pseudopotentials from the Quantum ESPRESSO database. A vacuum spacing of 18 Å was set along *c* direction to avoid unphysical interlayer interactions. The Brillouin zone was sampled by a fine *Γ*-centered 8 × 8 × 1 *k*-point Monkhorst–Pack grid.^54^

For the hybrid molecular/CrSBr heterostructures, we constructed 5 × 5 supercells of CrSBr to ensure a minimum distance of 10 Å between neighbouring molecules, thus avoiding spurious electrostatic interactions between them. In this case the Brillouin zone was sampled by a *Γ*-centered 3 × 3 × 1 *k*-point grid. Grimme-D2 dispersion corrections were added in order to account for vdW interactions between the organic molecules and the substrate.^55^ Note that the crystal lattice is not allowed to relax as we consider that the effect of a single molecule will not modify the lattice parameters of the substrate. The adsorption energy (*E*_ads_) was determined as *E*_ads_ = *E*_CrSBr+molecule_ − (*E*_CrSBr_ + *E*_molecule_), where *E*_CrSBr+molecule_, *E*_CrSBr_ and *E*_molecule_ are total energies of the hybrid heterostructure, pristine CrSBr and the isolated molecule, respectively. The energy levels of HOMO and CBM are referred to the vacuum energy for each system.

To calculate the HOMO and LUMO of the molecules, we have performed DFT calculations using the Gaussian09 programme package. We have employed UB3LYP functional^56^ along with Ahlrichs triple zeta valence plus polarization (TZVP) basis set for all the atoms in our calculations.^57^ The quadratic convergence method was employed throughout all the calculations. The effect of CrSBr on the HOMO and LUMO of the molecule has been estimated by considering a small supercell of CrSBr below the molecules to reduce the computational cost. Here we have used Ahlrichs triple zeta valence plus polarisation (TZVP) basis set for Cr, and split valence plus polarisation (SVP) basis set for all other atoms.

We estimated the CDD by subtracting the charge densities of the isolated components (molecules and pristine CrSBr monolayer) from the total charge density of the combined CrSBr + organic molecule system by applying the following formula:

1 Δ*ρ* = *ρ* (CrSBr + molecule) − *ρ* (CrSBr) − *ρ* (molecule)

where *ρ* (CrSBr + molecule) and *ρ* (CrSBr) represent the total electron densities of CrSBr monolayer with and without adsorbed molecule, respectively, and *ρ* (molecule) represents the electron density of the isolated molecule. It should be noted that the separate molecule and substrate must have the same distorted geometry as in the hybrid system.

The three magnetic exchange interactions (*J*_1_, *J*_2_ and *J*_3_) were extracted by mapping total energies from different magnetic configurations on a classical Heisenberg Hamiltonian. For such purpose we constructed 6 × 6 supercells to account for the magnetic pattern of the four magnetic configurations. In this case we considered *U*_eff_ = 3 eV (*U* = 4 eV and *J*_H_ = 1 eV, where *U*_eff_ = *U* − *J*_H_), in agreement with previous works.^58^ The *T*_C_ was obtained by performing atomistic simulations as implemented in the VAMPIRE code.^59^

Magnon dynamics is studied under the linear approximation of the Holstein–Primakoff boson expansion details are described in the ESI.†

## Author contributions

A. M. R and G. R. C contributed equally. All authors have read and agreed to the published version of the manuscript. All authors have given approval to the final version of the manuscript.

## Conflicts of interest

There are no conflicts to declare.

## Supplementary Material

NA-006-D4NA00230J-s001
