# Supplementary material for: Towards molecular controlled magnonics
Source: Nanoscale Adv. 2024 May 1;6(13):3320–8. doi: 10.1039/d4na00230j (PMC11197403; doi:10.1039/d4na00230j)
Supplement: NA-006-D4NA00230J-s001 [file NA-006-D4NA00230J-s001.pdf]

Electronic Supplementary Material (ESI) for Nanoscale Advances.
This journal is © The Royal Society of Chemistry 2024

Supporting Information

Towards molecular controlled magnonics

Alberto M. Ruiz,[a] Gonzalo Rivero-Carracedo,[a] Andrey Rybakov,[a]
Sourav Dey[a] and José J. Baldoví[*a]

[a]Instituto de Ciencia Molecular, Universitat de València, Catedrático José Beltrán 2,

46980 Paterna, Spain. E-mail: j.jaime.baldovi@uv.es

Table of Contents

1.Adsorption energy 1
2.Electronic structure 5
3.Magnetic structure 16
  3.1 Model Hamiltonian 16
  3.2 Effects on the structure 18
  3.3 Charge transfer 19
4. Magnons 23
  4.1 Magnon dispersion 23
  4.2 Group velocities 27
5. Bibliography 30

1.Adsorption energy

First, we test the stability of the heterostructures formed by CrSBr monolayer and the organic species TTF, perylene, coronene, and TCNQ by analysing their respective optimized geometries. To do so, we calculate the adsorption energy by applying the following expression:

$$E_{ads} = E_{CrSBr+molecule} - (E_{CrSBr} + E_{molecule}) \qquad (1)$$

where $E_{CrSBr+molecule}$, E_{CrSBr} and $E_{molecule}$ are the total energies of the hybrid molecular/2D heterostructure, pristine CrSBr and the isolated molecule, respectively. More negative adsorption energies indicate more stable heterostructures.

Table S1. Adsorption energy values for the different sites. Values in bold indicate the preferential site for adsorption.

	Adsorption energy, eV			
Site	TTF	Perylene	Coronene	TCNQ
Br-top	-1.39	**-1.35**	-1.09	**-0.65**
Cr-top	-1.45	-1.23	**-1.18**	-0.57
Hollow-top	**-1.52**	-1.25	-1.11	-0.59
S-top	-1.40	-1.18	-1.08	-0.59

Then, we study in plane rotations of the molecules in the previously determined preferential site to explore if the interaction strength can be increased by acquiring a different orientation (**Fig. S1**).

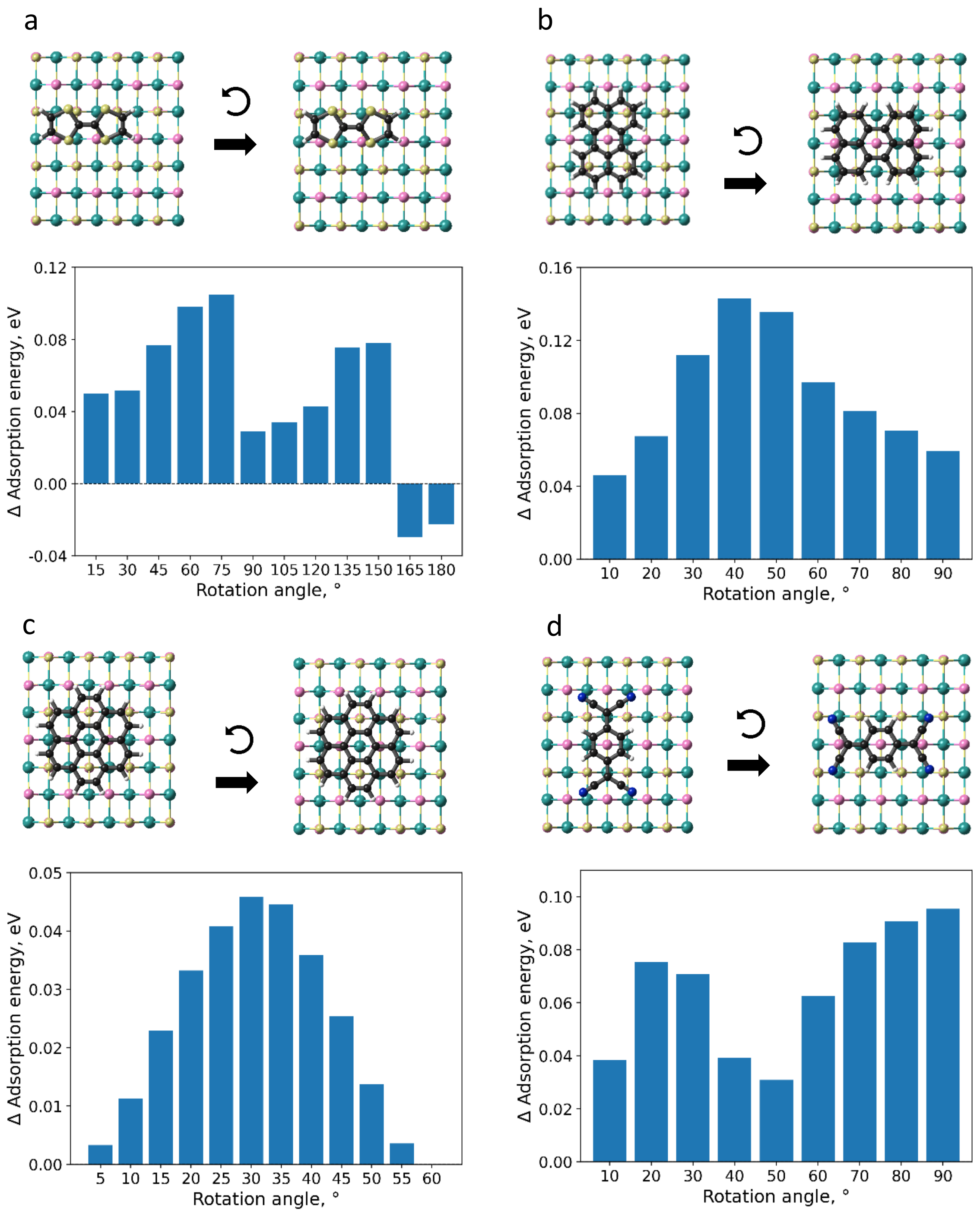

Fig. S1. Evolution of the adsorption energy for the selected rotations according to symmetry matching between molecule and substrate. (a) TTF, (b) perylene, (c) coronene, (d) TCNQ. Note that TTF was rotated 180° instead of 90° as for the rest of the C_{2v} symmetry molecules because of its tilted configuration.

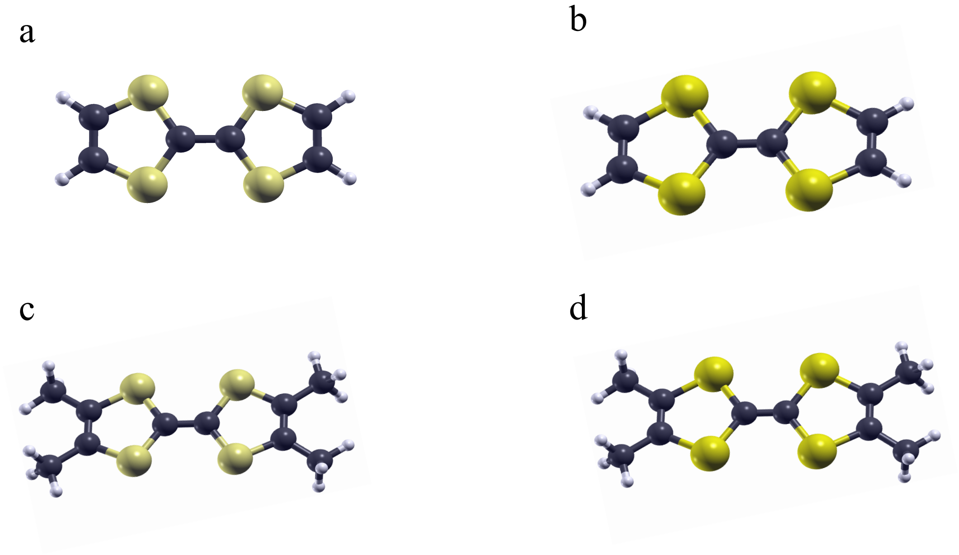

Fig. S2. Chemical structure of (a) TTF, (b) TSF, (c) TTF-CH_3 and (d) TSF-CH_3. Pale (vivid) yellow balls correspond to S (Se) atoms.

Table S2. Preferential site and adsorption energy for TTF and derivatives.

Molecules	Site	E_{ads}, eV
TTF	Hollow-top	-1.55
TSF	Hollow-top	-1.58
TTF-CH_3	Hollow-top	-2.23
TSF-CH_3	Hollow-top	-2.20

Table S3. Preferential site, adsorption energy, distance to the surface and charge transfer for the most stable configurations. Regarding electron transfer, positive (negative) sign represents charge donation (acceptance) to (from) the substrate.

Molecules	Site	E_{ads}, eV	h, Å	e^- transfer
TTF-CH_3	Hollow-top	-2.23	1.32	0.80
TTF	Hollow-top	-1.55	2.14	0.69
Perylene	Br-top	-1.35	2.76	0.45
Coronene	Cr-top	-1.18	2.97	0.16
TCNQ	Br-top	-0.65	3.15	-0.03

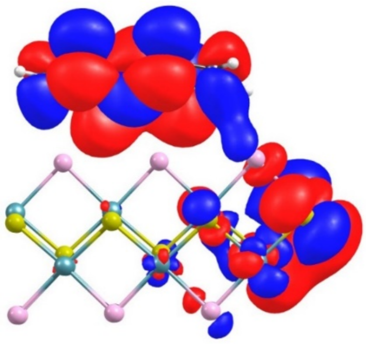

Fig. S3. HOMO orbital of TTF molecule adsorbed on CrSBr. Blue (red) surfaces represent positive (negative) values of the molecular orbital. Isovalue is set to 0.01 eV·Å^{-3}. The tilting of TTF can be attributed to the favourable overlap between the highest occupied molecular orbital (HOMO) and the π orbitals of the close Br atoms from the substrate, thus intensifying the binding strength.

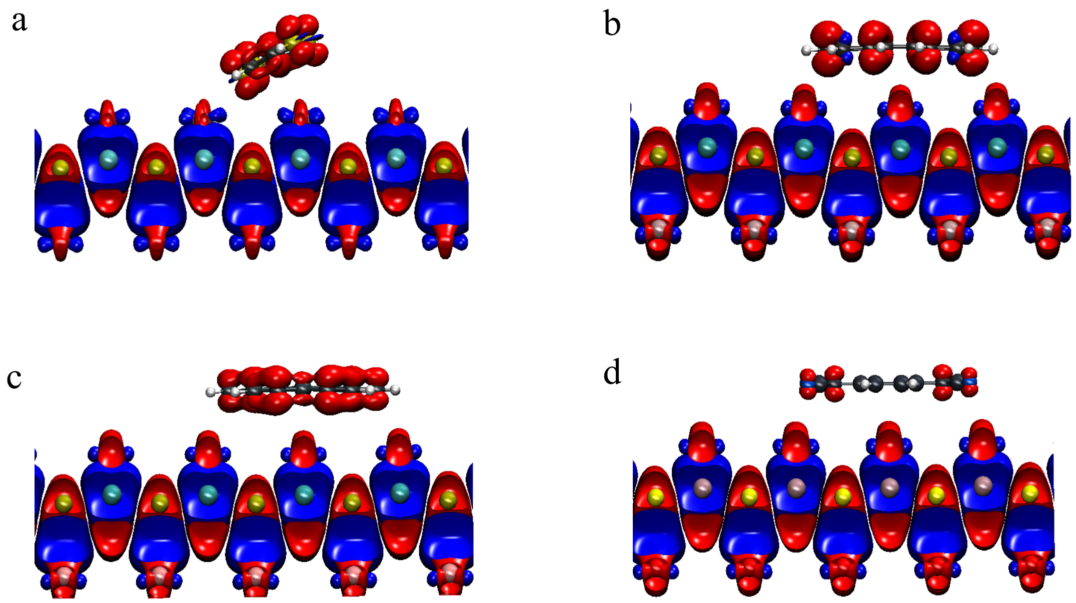

Fig. S4. Spin density distribution of the molecular/CrSBr hybrid heterostructures. It can be observed that the molecules become spin polarized after the adsorption, with a magnetic moment value of -0.24, -0.12, -0.08 and -6·10^{-4} μ_B for (a) TTF, (b) perylene, (c) coronene and (d) TCNQ, respectively. The isovalue is set to 0.001 eV·Å^{-3}.

2.Electronic structure

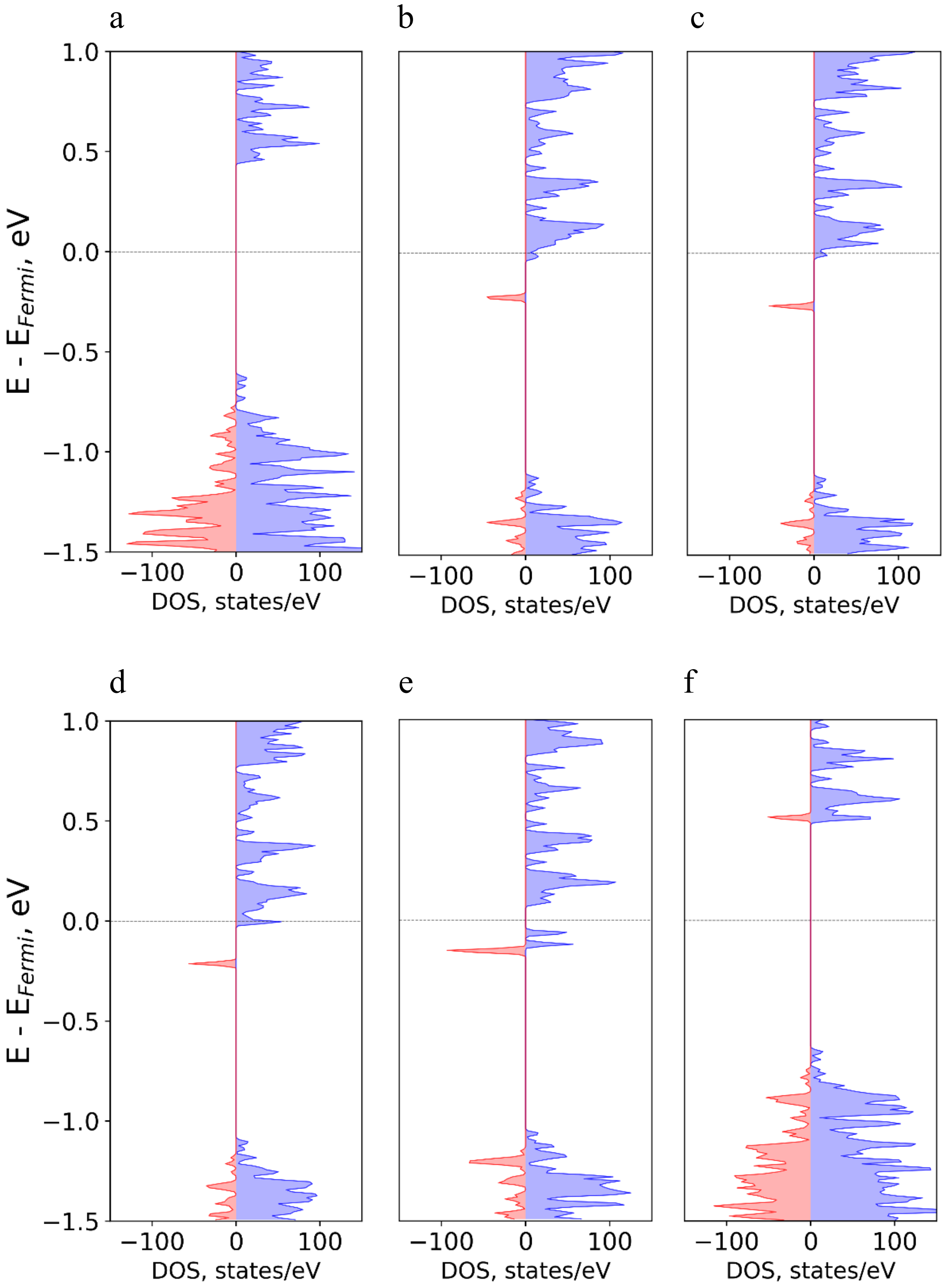

Fig. S5. Calculated density of states (DOS) for (a) pristine CrSBr and the hybrid molecular/CrSBr heterostructures based on (b) TTF-CH_3, (c) TTF, (d) perylene, (e) coronene, (f) TCNQ. Blue (red) colour represent spin up (down) states. For the donor molecules, note that the new highly localized states between valence and conduction bands appear close to Fermi energy (E_F). Donors push E_F towards the conduction bands.

Table S4. Vacuum energy (E_{vac}), Fermi energy (E_{Fermi}) and work function (Φ) for pristine CrSBr and adsorption systems. E_F increases and Φ decreases with the donor behaviour of molecules, respectively. Work function is calculated by: $\Phi = E_{vac} - E_F$.

	E_{vac}, eV	E_{Fermi}, eV	Φ, eV
CrSBr	4.00	-2.26	6.26
TTF-CH_3	3.49	-1.42	4.91
TTF	3.52	-1.51	5.03
Perylene	3.65	-1.54	5.19
Coronene	3.75	-1.62	5.37
TCNQ	3.74	-2.15	5.89

Next, we show the projected density of states (PDOS) of the Cr atoms of the substrate for the pristine and different adsorption systems. In addition, we show the DOS for the isolated molecules and adsorbed molecules. In the latter case we observe the splitting of the molecular states for the different cases (all **Figures** are plotted with RAD-tools[1]).

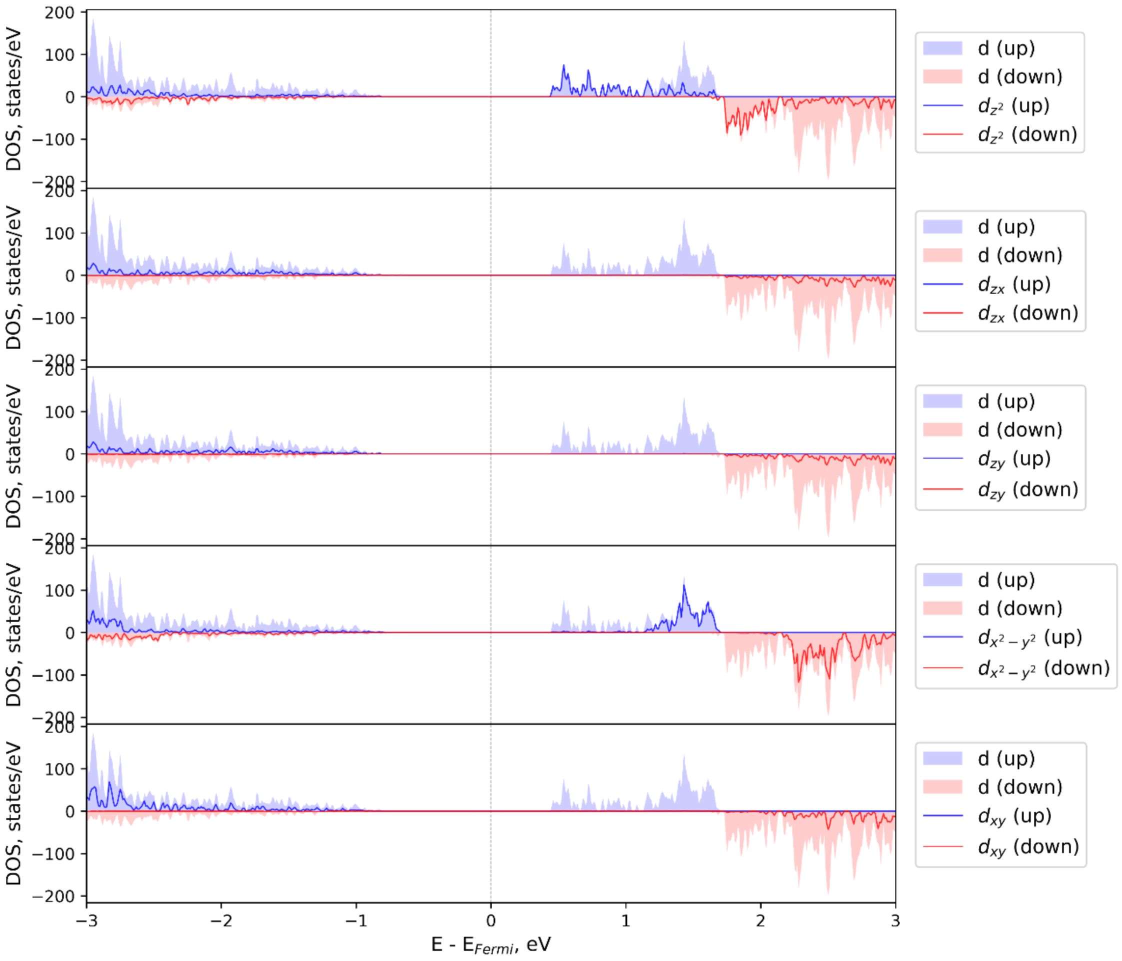

Fig.S6. Calculated projected density of states (PDOS) for the Cr d orbitals in pristine CrSBr.

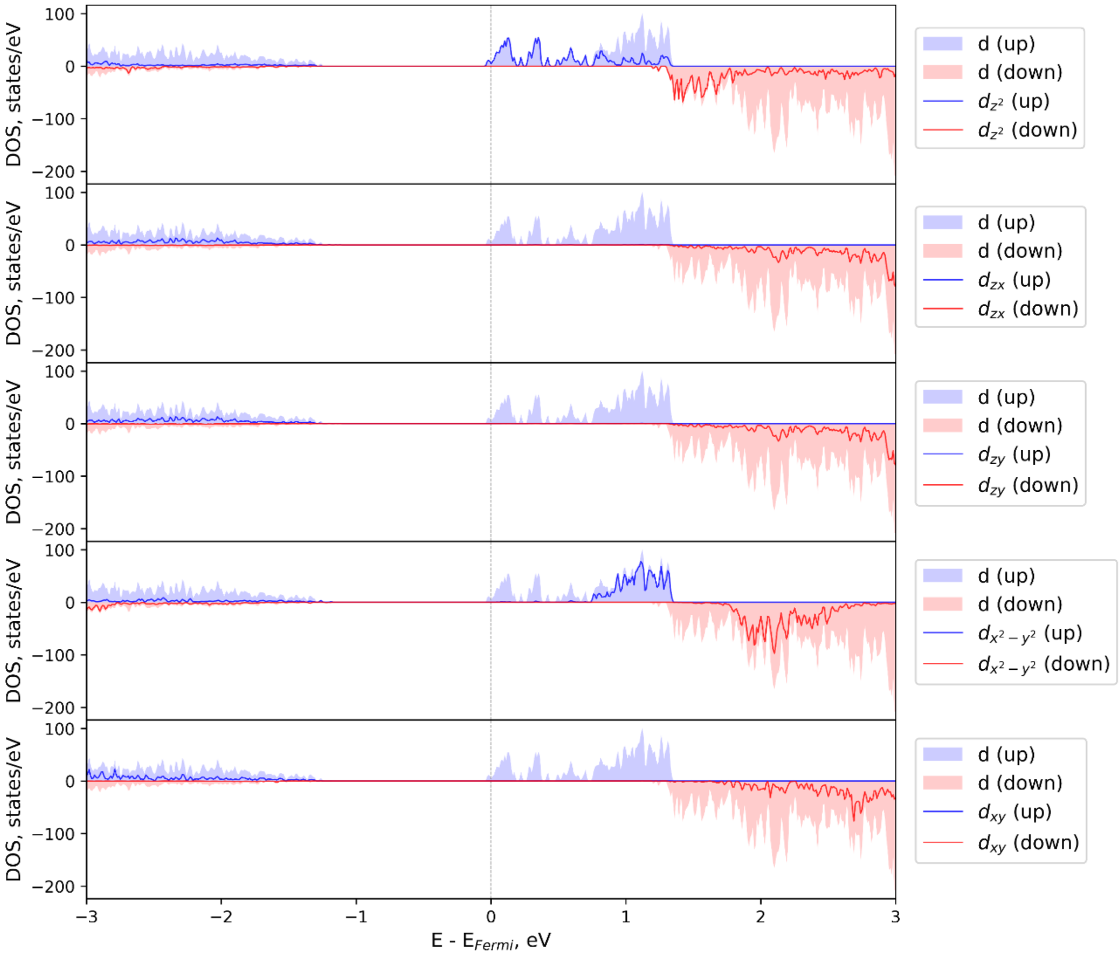

Fig. S7. Calculated PDOS for the Cr d orbitals in the hybrid TTF-CH_3/CrSBr heterostructure.

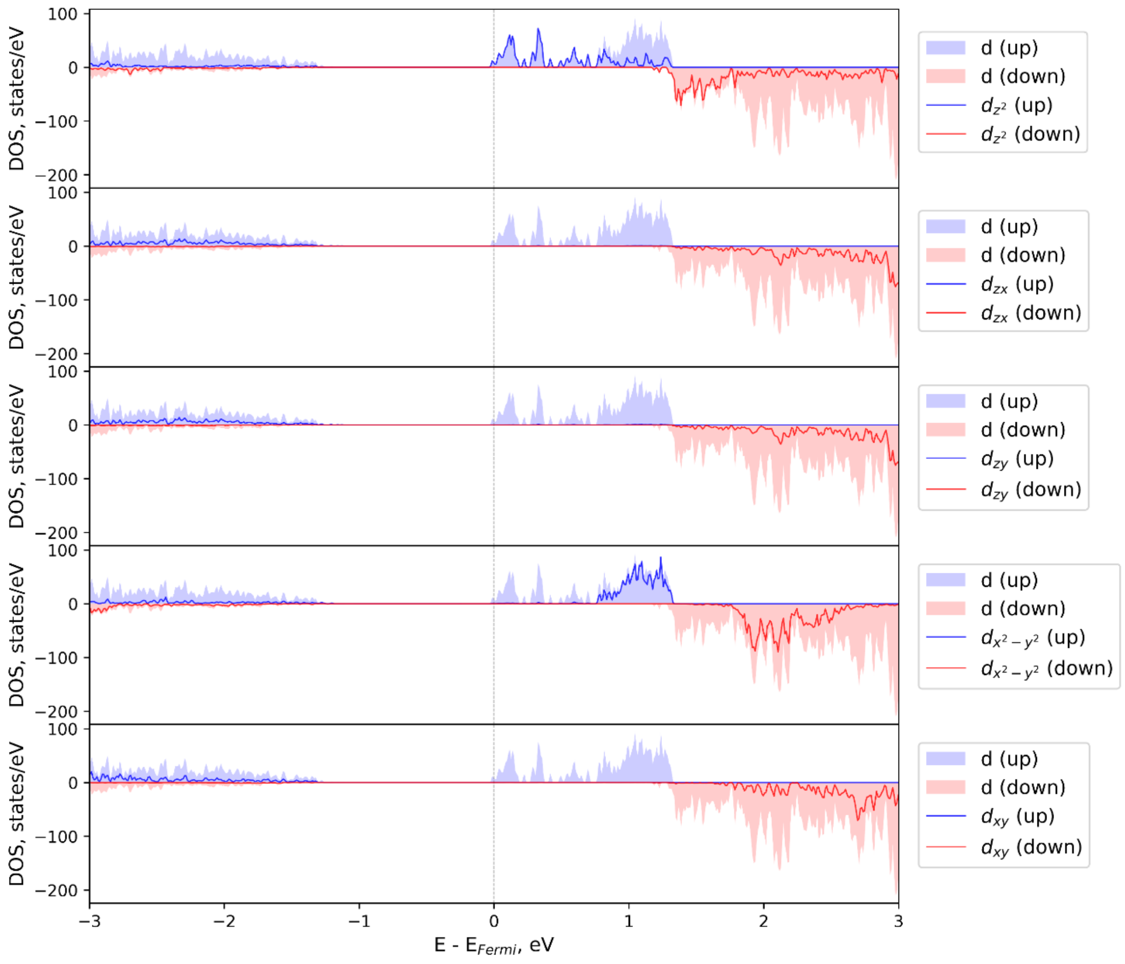

Fig. S8. Calculated PDOS for the Cr d orbitals in the hybrid TTF/CrSBr heterostructure.

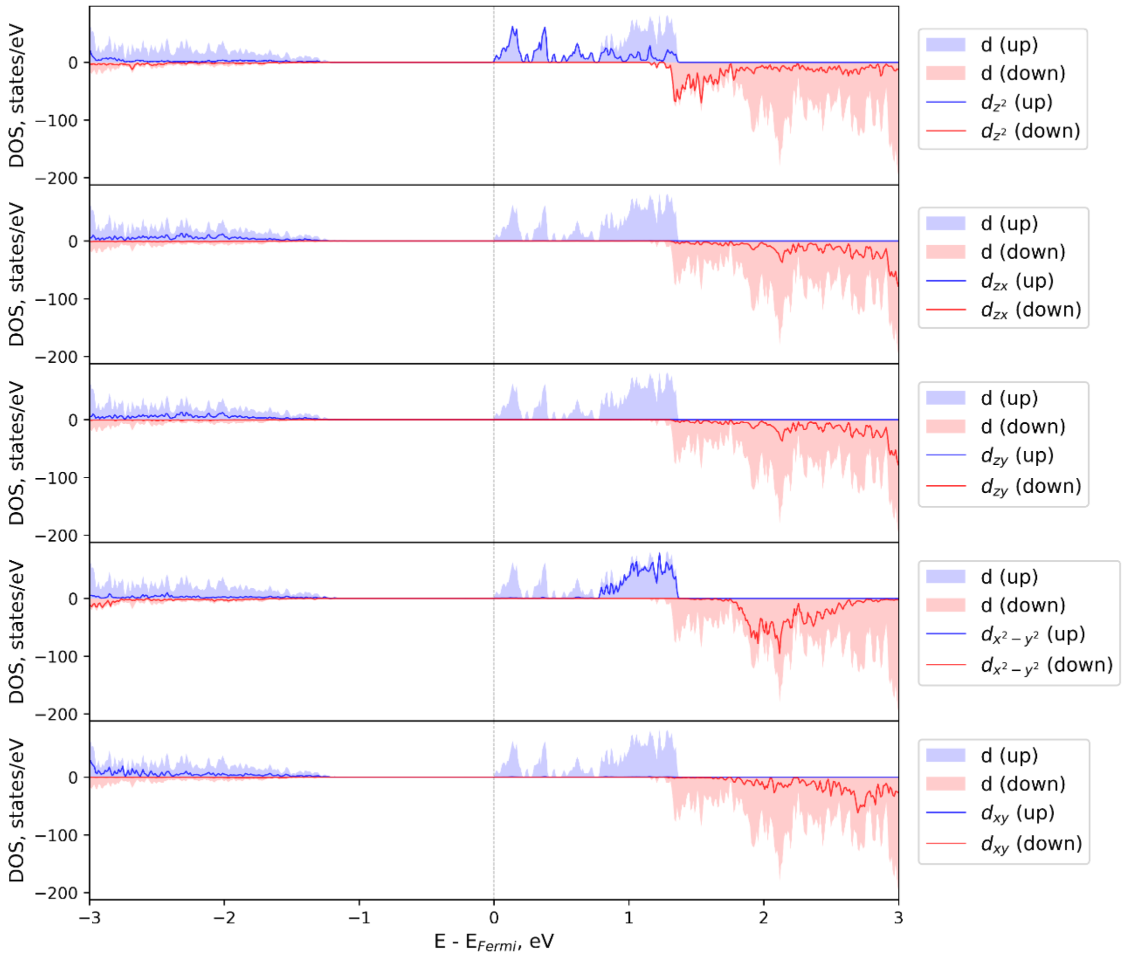

Fig. S9. Calculated PDOS for the Cr d orbitals in the hybrid perylene/CrSBr heterostructure.

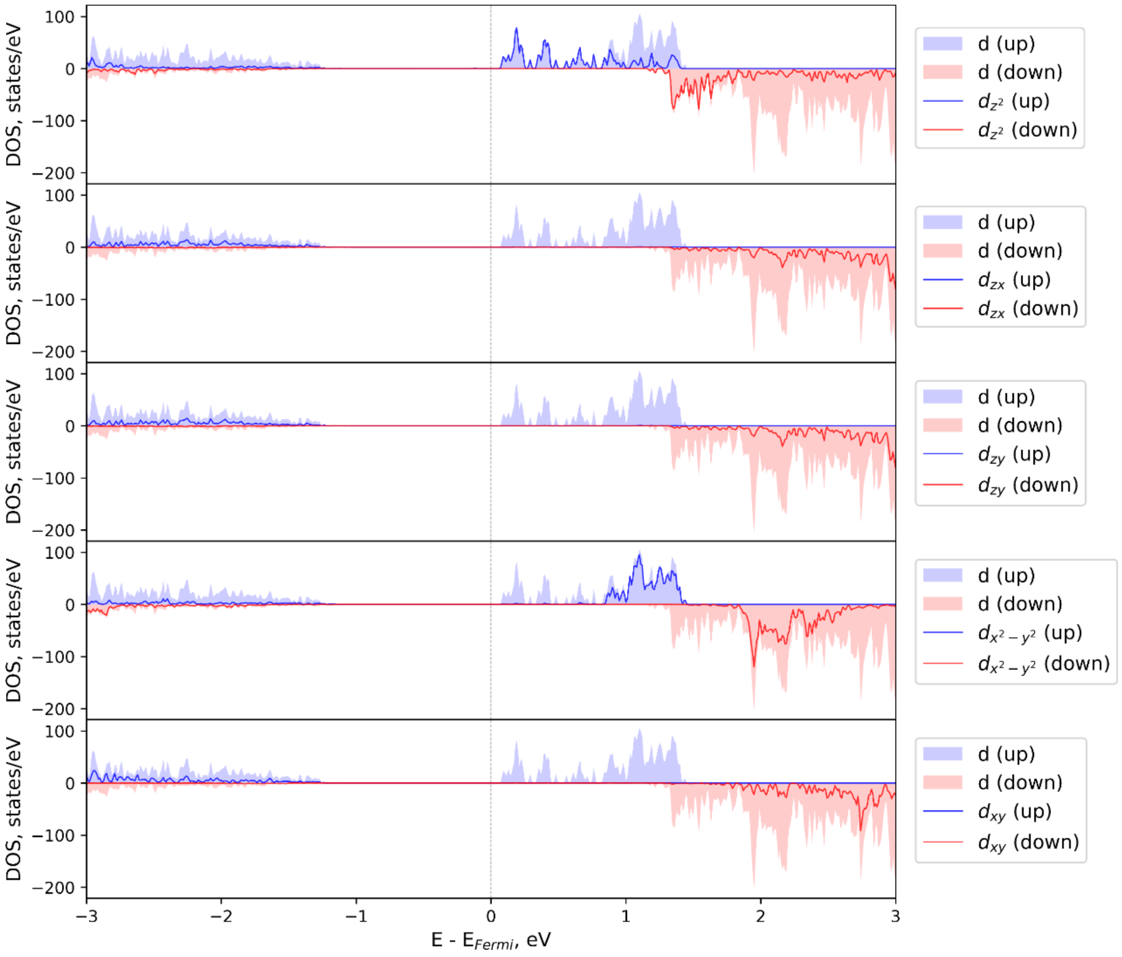

Fig. S10. Calculated PDOS for the Cr d orbitals in the hybrid coronene/CrSBr heterostructure.

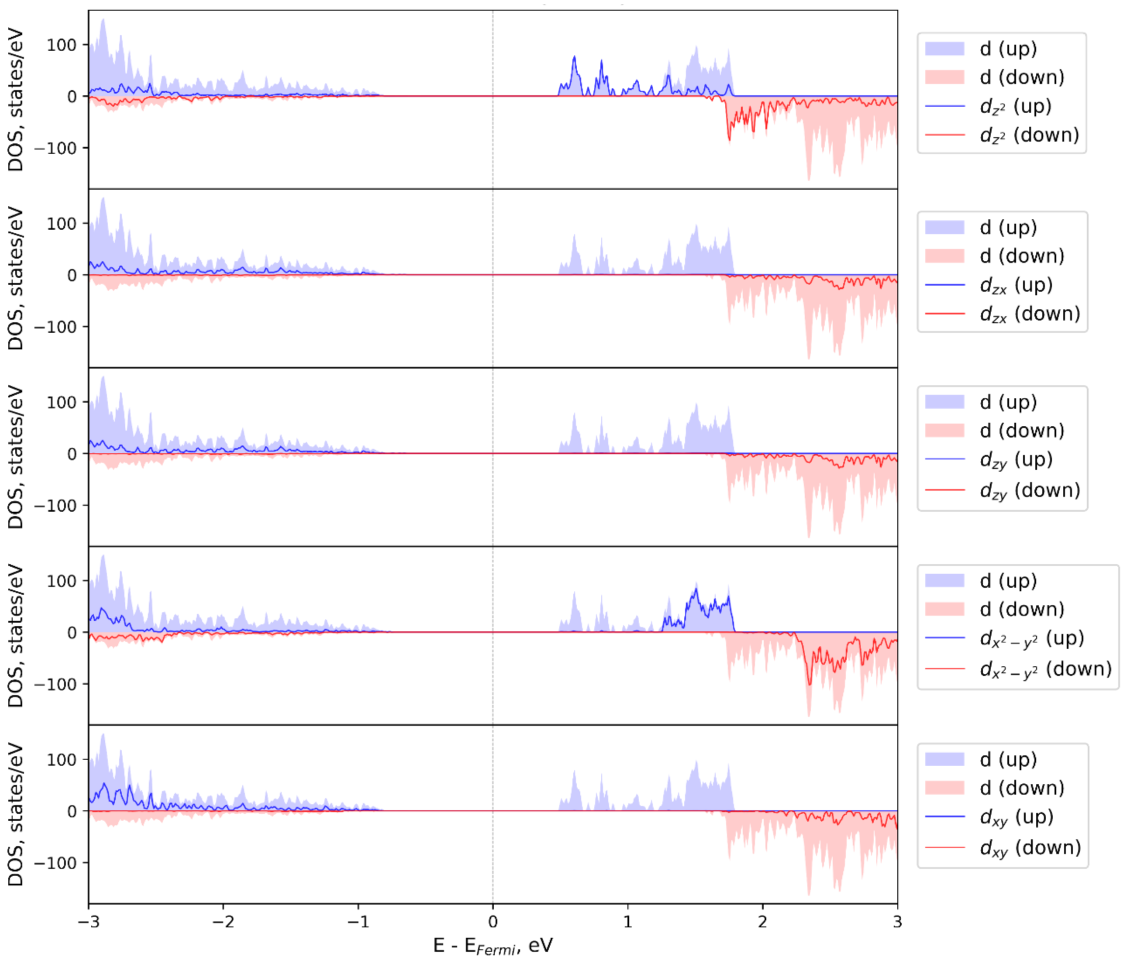

Fig. S11. Calculated PDOS for the Cr d orbitals in the hybrid TCNQ/CrSBr heterostructure.

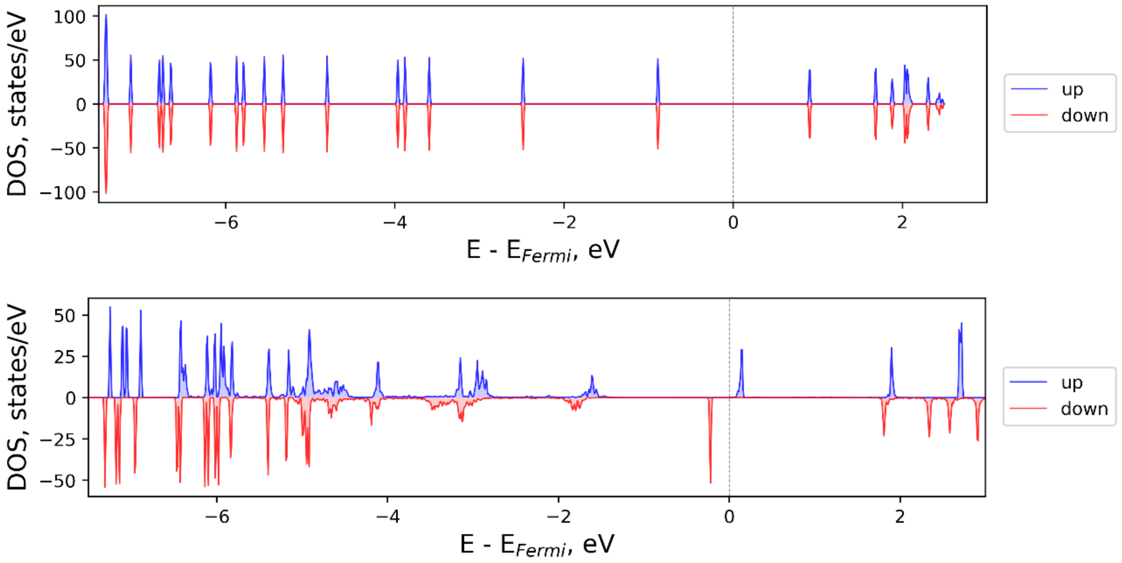

Fig. S12. Calculated DOS of free (top) and adsorbed-distorted (bottom) TTF-CH_3 molecule.

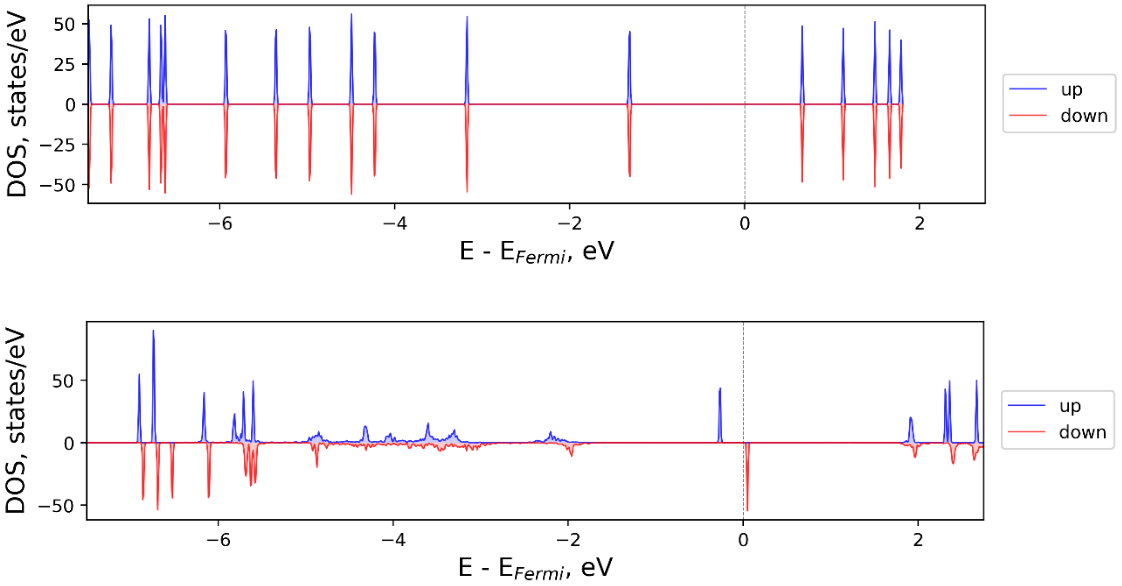

Fig. S13. Calculated DOS of free (top) and adsorbed-distorted (bottom) TTF molecule.

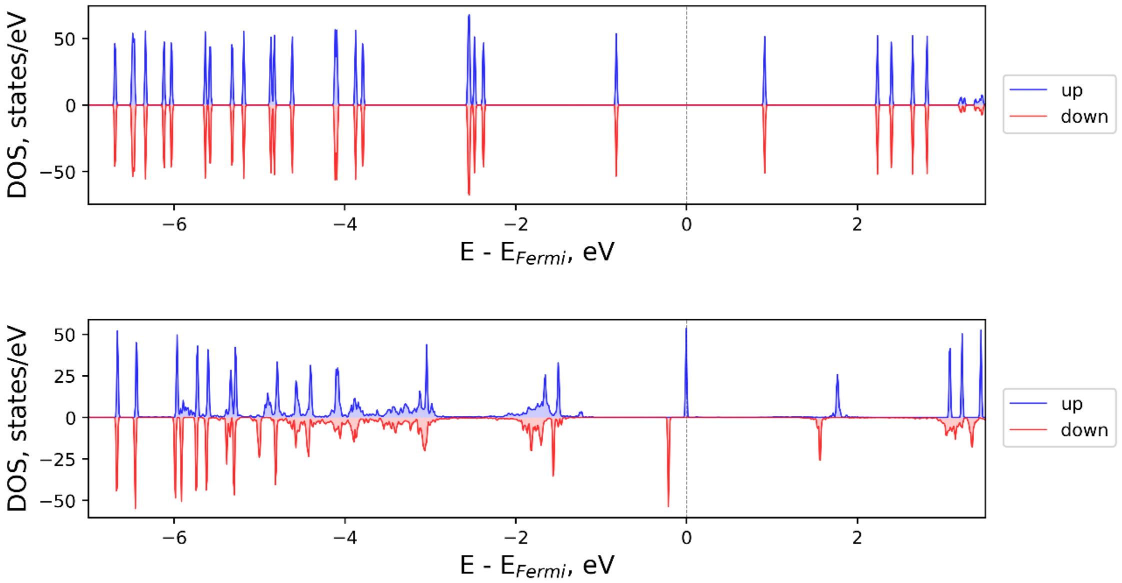

Fig. S14. Calculated DOS of free (top) and adsorbed-distorted (bottom) perylene molecule.

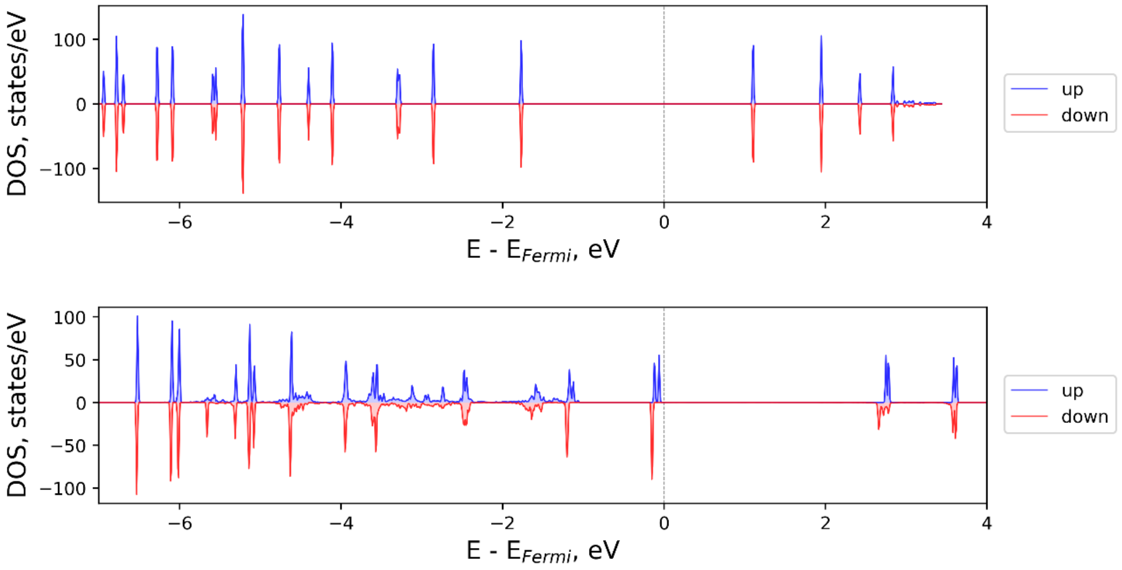

Fig. S15. Calculated DOS of free (top) and adsorbed-distorted (bottom) coronene molecule.

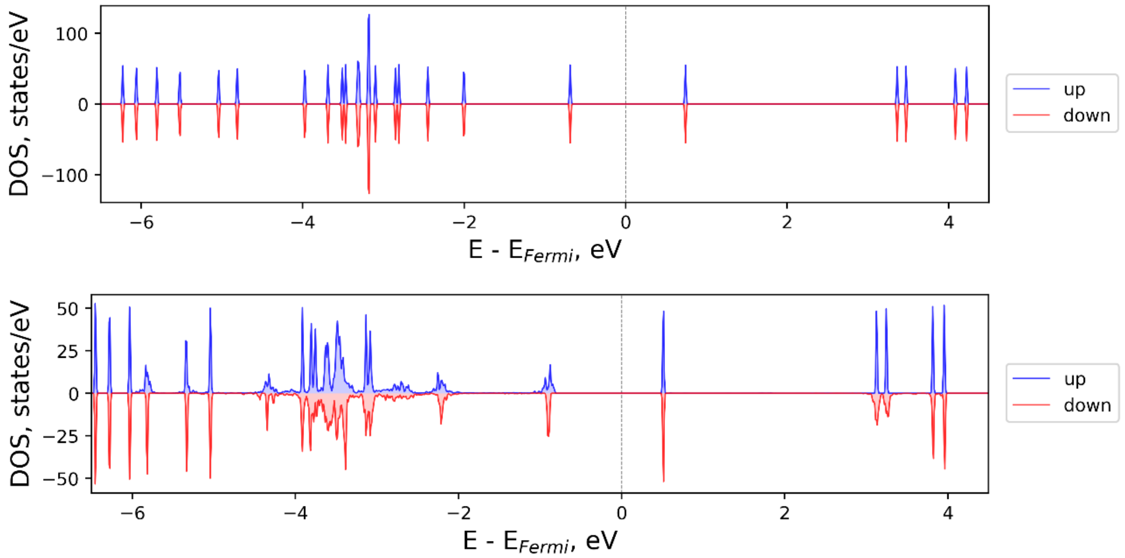

Fig. S16. Calculated DOS of free (top) and adsorbed-distorted (bottom) TCNQ molecule.

3.Magnetic structure

3.1 Model Hamiltonian

Magnetic properties of the CrSBr are modelled with the Hamiltonian of the form:

$$\hat{H} = -\sum_{ij} \hat{S}_i J_{ij} \hat{S}_j$$

where double counting is present and spins are normalized to 1. In this study we investigate the effect of the molecule deposition on the isotropic part of exchange interaction. The exchange parameters are computed via energy mapping with the four configurations displayed in **Fig. S17**.

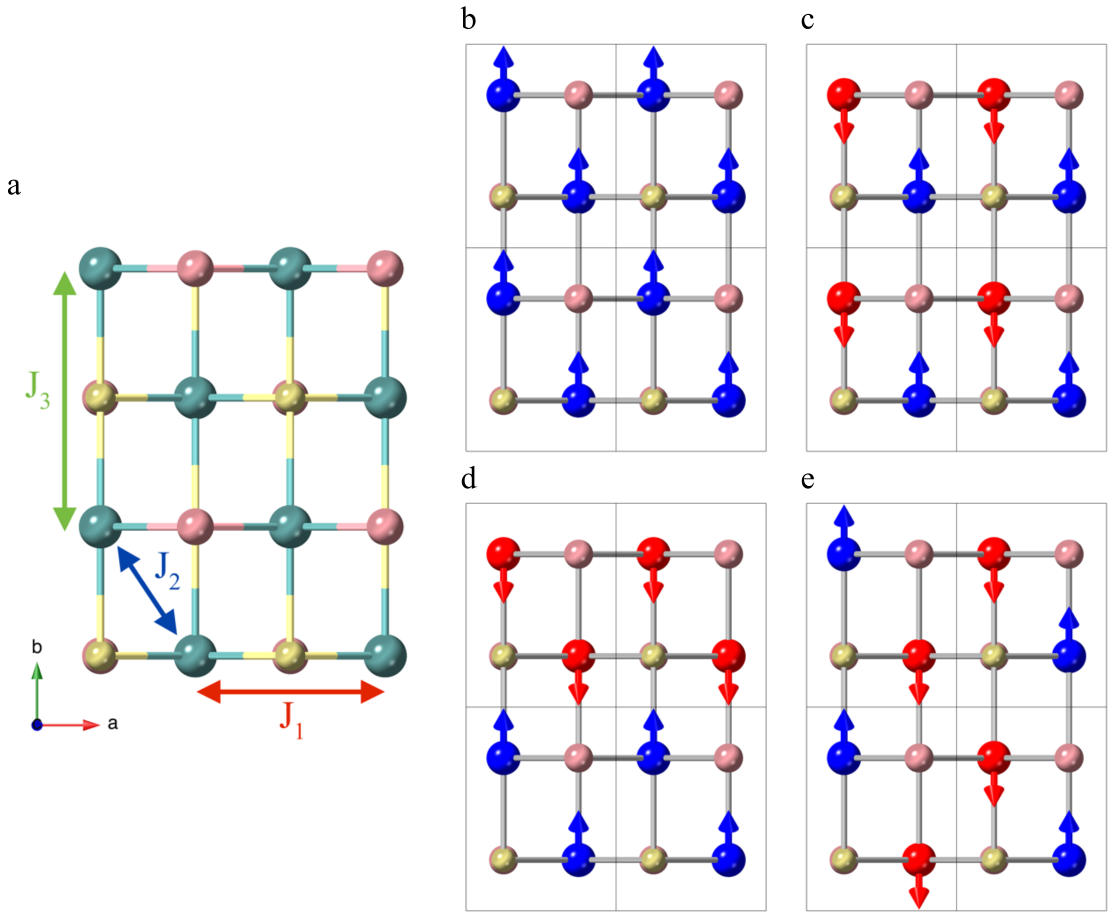

Fig. S17. (a) CrSBr monolayer marked with the three nearest neighbours exchange parameters depicted as J_1, J_2 and J_3. On the right, the four computed magnetic configurations: (b) FM (c) AF_1 (d) AF_2 (e) AF_3. Blue and red arrows represent Cr atoms with spin up and spin down state, respectively. Thin black lines indicate the unit cells.

Exchange parameters are computed as:

$$J_2 = \frac{E_{AF_1} - E_{FM}}{8}$$
$$J_1 = J_2 + \frac{E_{AF_3} - E_{AF_1}}{4}$$
$$J_3 = J_2 + \frac{E_{AF_2} - E_{AF_1}}{4}$$

where E_{FM}, E_{AF_1}, E_{AF_2}, E_{AF_3} - are the energies of the corresponding configurations per one Cr atom. The computed values of the total energies and computed exchange parameters are listed in **Table S5.**

Table S5. Relative total energies ΔE (meV/ Cr atom) for the four magnetic configurations scaled with respect the energy of ferromagnetic configuration. The three magnetic exchange parameters J_1, J_2 and J_3 (meV) are listed. Data for the calculations with molecules on top of CrSBr.

Molecules	ΔE_{FM}	ΔE_{AF_1}	ΔE_{AF_2}	ΔE_{AF_3}	J_1	J_2	J_3
CrSBr	0	30.97	26.51	27.94	3.11	3.87	2.76
TTF-CH_3	0	31.20	28.29	28.93	3.33	3.90	3.17
TTF	0	31.11	27.23	28.77	3.30	3.89	2.92
Perylene	0	30.99	25.21	28.41	3.23	3.87	2.43
Coronene	0	30.83	23.68	28.11	3.17	3.85	2.06
TCNQ	0	30.70	23.66	27.96	3.15	3.84	2.08

3.2 Effects on the structure

In order to describe the structural distortion of the CrSBr substrate caused by the deposited molecule we define the characteristic angles and bond lengths. The angles are depicted in the Fig. S18. Three distances are defined as the distances between two nearest Cr atoms along the direction of the first lattice vector *a*, second lattice vector *b* and between two atoms in the unit cell (ab). The values are presented in Table S7.

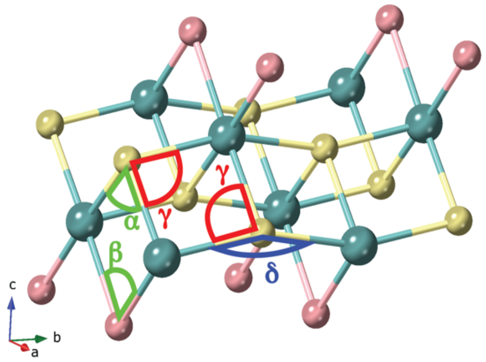

Fig. S18. Characteristic angles of CrSBr structure. The Cr, S and Br atoms are represented as cyan, yellow and pink spheres, respectively.

Table S6. Relative total energies ΔE (meV/ Cr atom) for the four magnetic configurations scaled with respect the energy of ferromagnetic configuration. The three magnetic exchange parameters J_1, J_2 and J_3 (meV) are listed. Data for the calculations with the distortion in the CrSBr substrate, caused by each molecule, but without the molecules.

Distortion	ΔE_{FM}	ΔE_{AF_1}	ΔE_{AF_2}	ΔE_{AF_3}	J_1	J_2	J_3
CrSBr	0	30.97	26.51	27.94	3.11	3.87	2.76
TTF-CH_3	0	30.76	23.00	27.93	3.14	3.84	1.90
TTF	0	30.74	23.02	27.95	3.14	3.84	1.91
Perylene	0	30.77	23.24	27.95	3.14	3.85	1.96
Coronene	0	30.75	23.30	27.94	3.14	3.84	1.98
TCNQ	0	30.70	23.67	27.95	3.15	3.84	2.08

Table S7. Structural parameters. Bond lengths and angles between the specified atoms. They represent the average values where the considered Cr atoms in the heterostructures are the ones located in the substrate area that is under the molecule. Notation is referred to Fig. S18.

Angle	CrSBr	TTF-CH_3	TTF	Perylene	Coronene	TCNQ
Cr-Cr (a), Å	3.60	3.61	3.60	3.61	3.60	3.60
Cr-Cr (b), Å	4.82	4.84	4.83	4.83	4.82	4.82
Cr-Cr (ab), Å	3.65	3.62	3.63	3.62	3.63	3.64
Cr-S-Cr (α), °	95.76	95.53	95.88	96.03	95.98	96.00
Cr-Br-Cr (β), °	89.66	89.24	89.08	90.08	89.68	90.00
Cr-S-Cr (γ), °	97.49	95.90	96.19	96.28	96.60	96.69
Cr-S-Cr (δ), °	157.59	162.71	161.94	161.78	160.27	159.96

3.3 Charge transfer

We study the charge transfer flowing along the interface between CrSBr monolayer and the molecules by performing a Bader analysis as stated in the main text. The charge donation to the substrate can be depicted in the population of the d_{z^2} orbital (Figs. S19, S20).

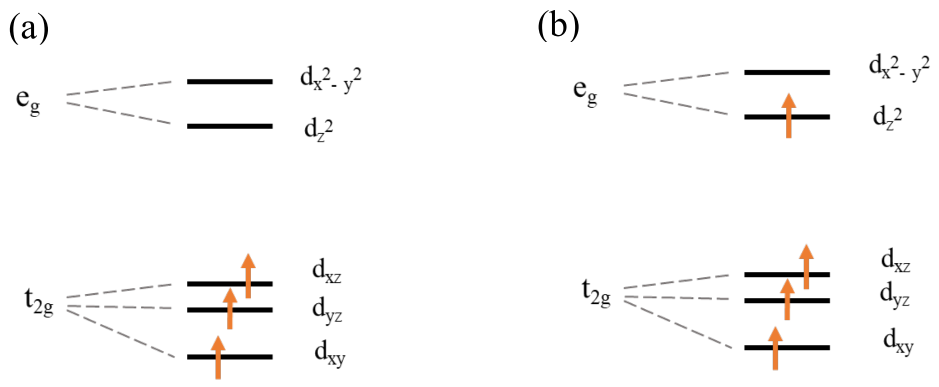

Fig. S19. Orbital splitting of Cr^{3+} ion in a distorted octahedron with C_{2v} symmetry. Orange arrows represent the electrons that are filling the Cr d orbitals in (a) pristine CrSBr and (b) donor molecule/CrSBr heterostructure.

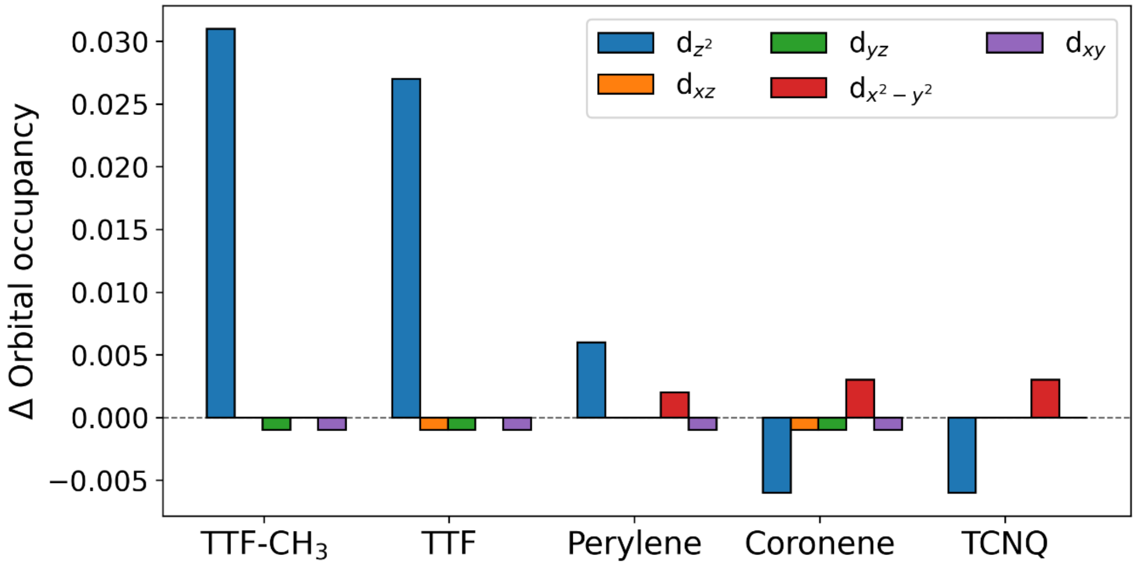

Fig. S20. Orbital occupancy of Cr d orbitals for the adsorption systems relative to pristine CrSBr. Note that the occupancy is referred to the averaged value of the Cr atoms that are located in the area covered by the molecule.

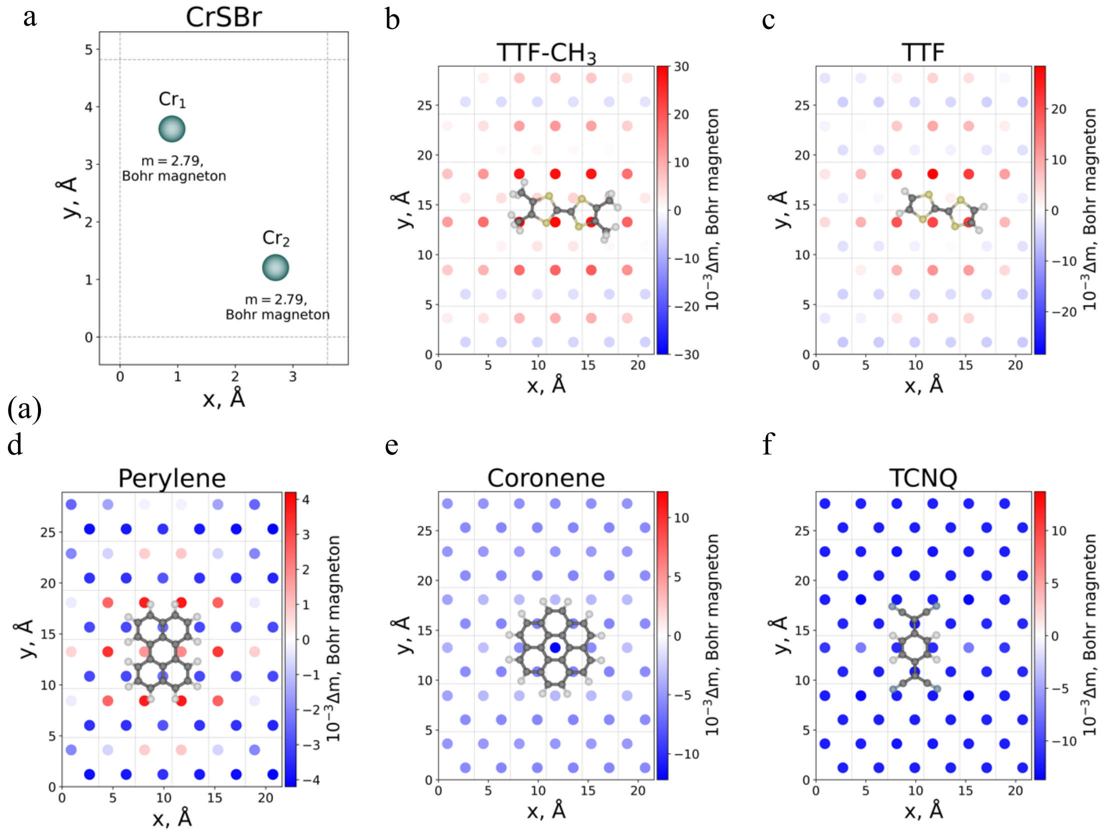

Fig. S21. Magnetic moments of Cr atoms (depicted as balls) in (a) pristine CrSBr substrate, and with the (b) TTF-CH$_3$, (c) TTF, (d) Perylene, (e) Coronene, (f) TCNQ molecule.

Table S8. Relative total energies ΔE (meV/ Cr atom) for the four magnetic configurations scaled with respect the energy of ferromagnetic configuration. The three magnetic exchange parameters J_1, J_2 and J_3 (meV) are listed. Data for the calculations with the distortion in the CrSBr substrate, caused by each molecule and corresponding electron doping.

Electron doping	ΔE_{FM}	ΔE_{AF_1}	ΔE_{AF_2}	ΔE_{AF_3}	J_1	J_2	J_3
CrSBr	0	30.97	26.51	27.94	3.11	3.87	2.76
TTF-CH_3	0	31.11	28.21	28.78	3.31	3.89	3.16
TTF	0	31.04	27.52	28.69	3.29	3.88	3.00
Perylene	0	30.98	26.18	28.44	3.24	3.87	2.67
Coronene	0	30.83	24.37	28.12	3.18	3.85	2.24
TCNQ	0	30.70	23.67	27.95	3.15	3.84	2.08

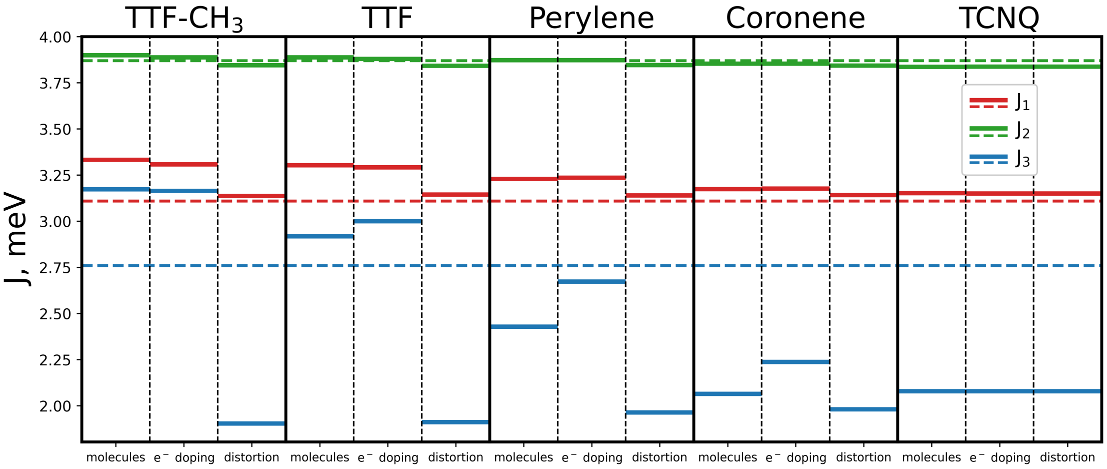

Fig. S22. Evolution of exchange parameters for the hybrid molecular/CrSBr heterostructures, electron doping and structural distortion. Values of the pristine CrSBr are shown with dashed lines.

When the molecules are deposited on top of the substrate, they change the magnetic moments of the Cr atoms. In the model Hamiltonian all changes are absorbed in the J values, however here we show the evolution of magnetic moments directly.

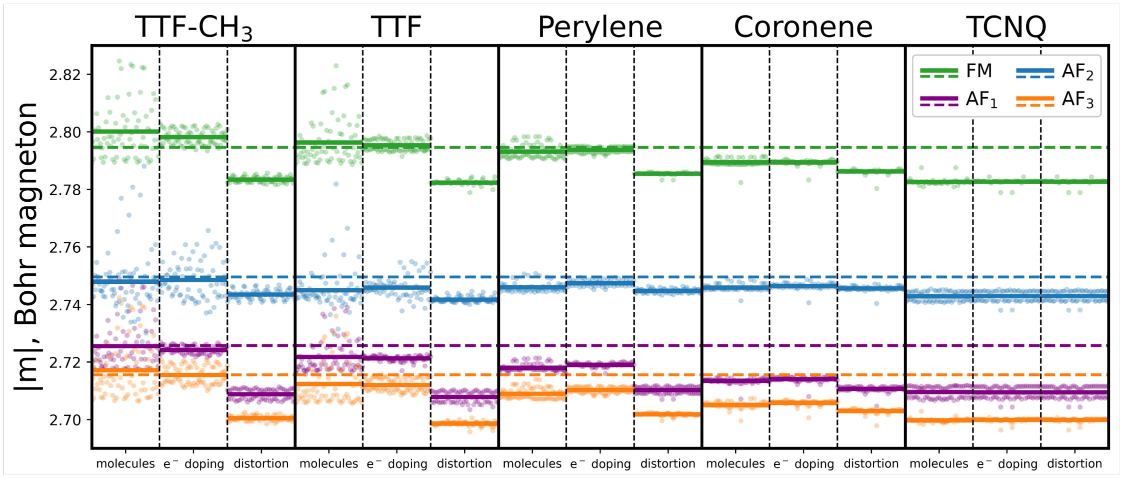

Fig. S23. Distribution of the magnetic moments for the 6x6 supercell subjected to the molecules, electron doping and structural distortion. Dots represent magnetic moments of each individual Cr atom. Solid lines show the mean value for each corresponding magnetic configuration. Dashed lines illustrate magnetic moments of each configuration for the pristine CrSBr.

Table S9. Relative total energies ΔE (meV/ Cr atom) for the four magnetic configurations scaled with respect the energy of ferromagnetic configuration. The three magnetic exchange parameters J_1, J_2 and J_3 (meV) are listed. Data corresponds to pristine CrSBr, TTF/CrSBr in a 6x6 supercell (low coverage) and TTF/CrSBr in a 4x2 supercell (high coverage).

	ΔE_{FM}	ΔE_{AF_1}	ΔE_{AF_2}	ΔE_{AF_3}	J_1	J_2	J_3
CrSBr	0	30.97	26.51	27.94	3.11	3.87	2.76
TTF-low coverage	0	31.04	27.52	28.69	3.29	3.88	3.00
TTF-high coverage	0	31.17	35.48	30.28	3.67	3.90	4.97

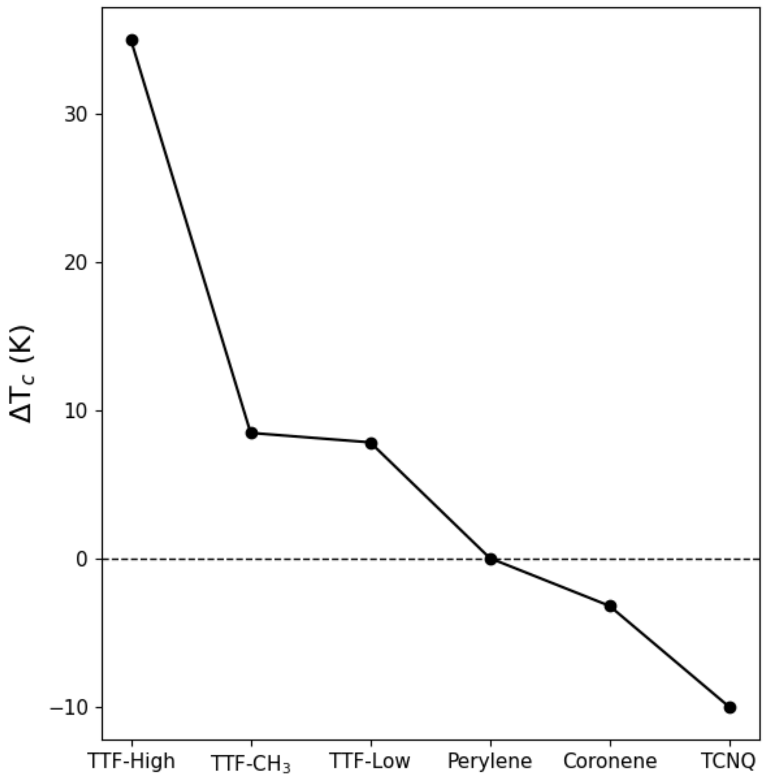

Fig. S24. Variation of the Curie Temperature (ΔT_C) for the different heterostructures with respect to pristine CrSBr (dashed line). The calculated T_C of monolayer CrSBr is 140 K.

4. Magnons

4.1 Magnon dispersion

Modelling of the magnon dispersion commences with the Hamiltonian, which differs from the model Hamiltonian (see section 3.1): spins are considered not to be normalized. In order to achieve that we map the supercell calculation to the simple model of the magnetic lattice of Cr atoms in the high spin state (3/2), effectively absorbing all effects in the J values themselves.

Magnons are defined as the excitation with respect to the ferromagnetic ground state, however in the model Hamiltonian there is no anisotropic terms, which are required to form ferromagnetic state in the 2D limit. As we stated before, more realistic Hamiltonian includes these terms, however they are not important to the analysis, which we are about to perform. Exchange and single-ion anisotropies contribute to the gap opening of the magnon dispersion effectively shifting the dispersion for the whole Brillouin zone, which does not affect the group velocities. Therefore, the evolution of the isotropic exchange is sufficient for the determination of the propagation velocities. The magnon dispersion relation is obtained via well-known Holstein-Primakoff (HP) transformation[2]:

$$\hat{S}_i^+ = \sqrt{2S}\hat{a}_i$$

$$\hat{S}_i^- = \sqrt{2S}\hat{a}_i^+$$
$$\hat{S}_i^z = S_i - \hat{a}_i^+\hat{a}_i$$
$$\omega(\boldsymbol{k}) = 2S\sum_i J_i n_i\left(1 - \gamma_{\boldsymbol{k}}^{(i)}\right)$$

where i runs in (1, 2, 3) for the nearest, next-nearest and next-next-nearest neighbors, n_i is the amount of the neighbors (2, 4, 2 correspondingly), $\gamma_{\boldsymbol{k}}^{(i)}$ are the structural factors:

$$\gamma_{\boldsymbol{k}}^{(1)} = \cos(k_x a)$$
$$\gamma_{\boldsymbol{k}}^{(2)} = \pm\cos\left(\frac{k_x a}{2}\right)\cos\left(\frac{k_y b}{2}\right)$$
$$\gamma_{\boldsymbol{k}}^{(3)} = \cos(k_y b)$$

where a and b are the lattice parameters; plus sign in the second structural factor corresponds to the acoustic (bottom) branch, minus sign refers to the optical (upper) branch.

In Fig. S25 we show a direct comparison between our calculations and the measured magnon spectrum for CrSBr.[3] As we can observe our theoretical predictions reproduce accurately the experimental data.

In Figs. S26 and S27 the magnon dispersion is presented (i) considering solely the distortion of the substrate caused by the molecules and (ii) distortion of the substrate and electron doping.

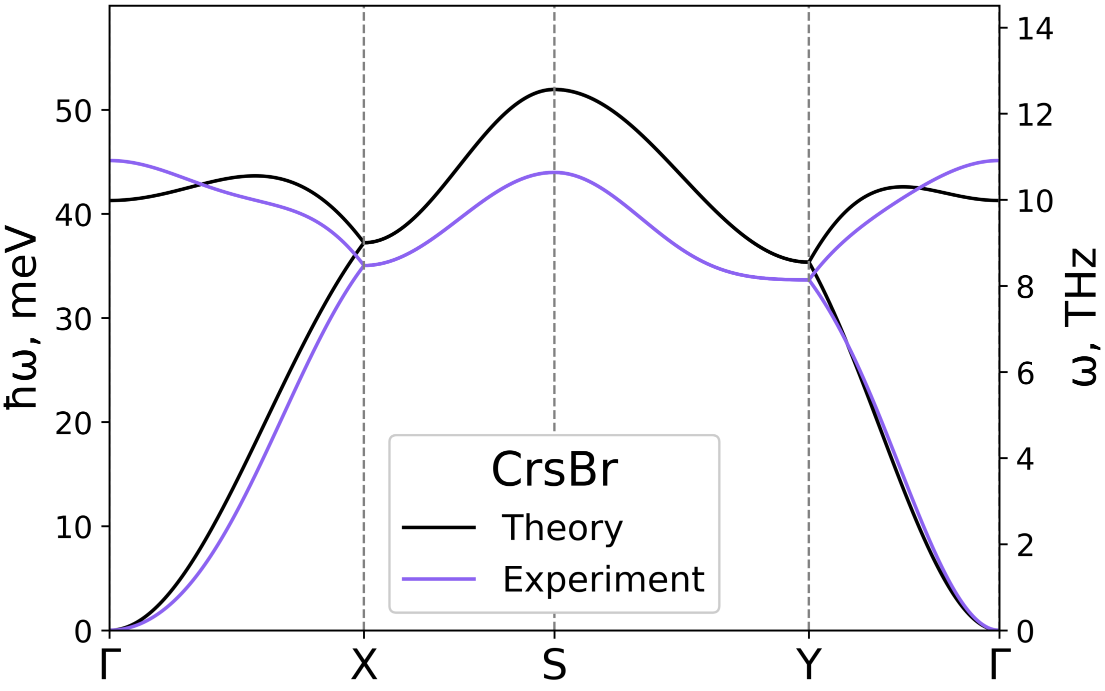

Fig. S25. Direct comparison between the magnon spectrum of CrSBr obtained from our calculations and the experimental one reported by Scheie et al.[3]

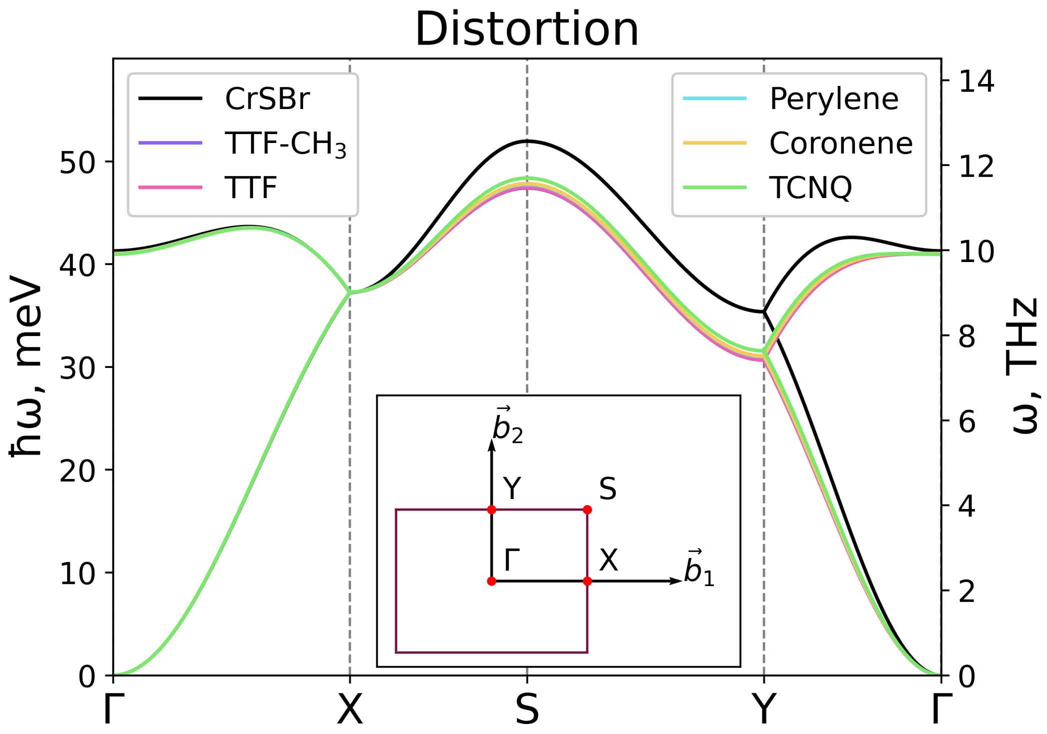

Fig. S26. Magnon dispersion for the structural distortions, caused by each molecule. Insert shows the k-path.

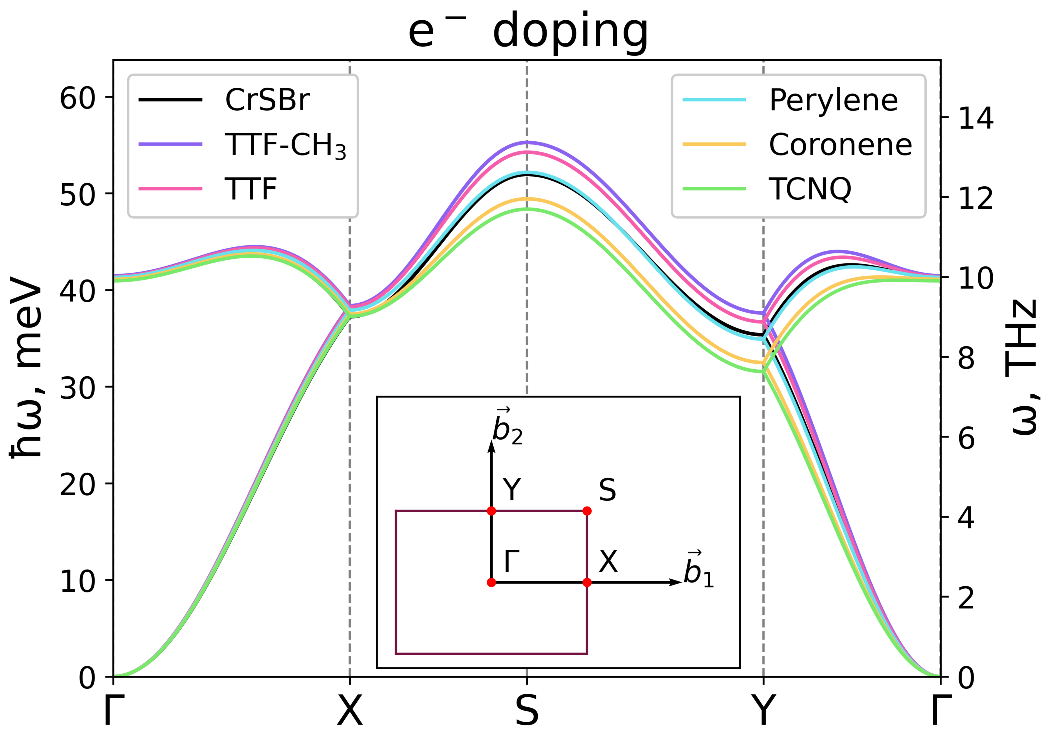

Fig. S27. Magnon dispersion for the structural distortions and electron doping corresponding to each molecule. Insert shows the k-path.

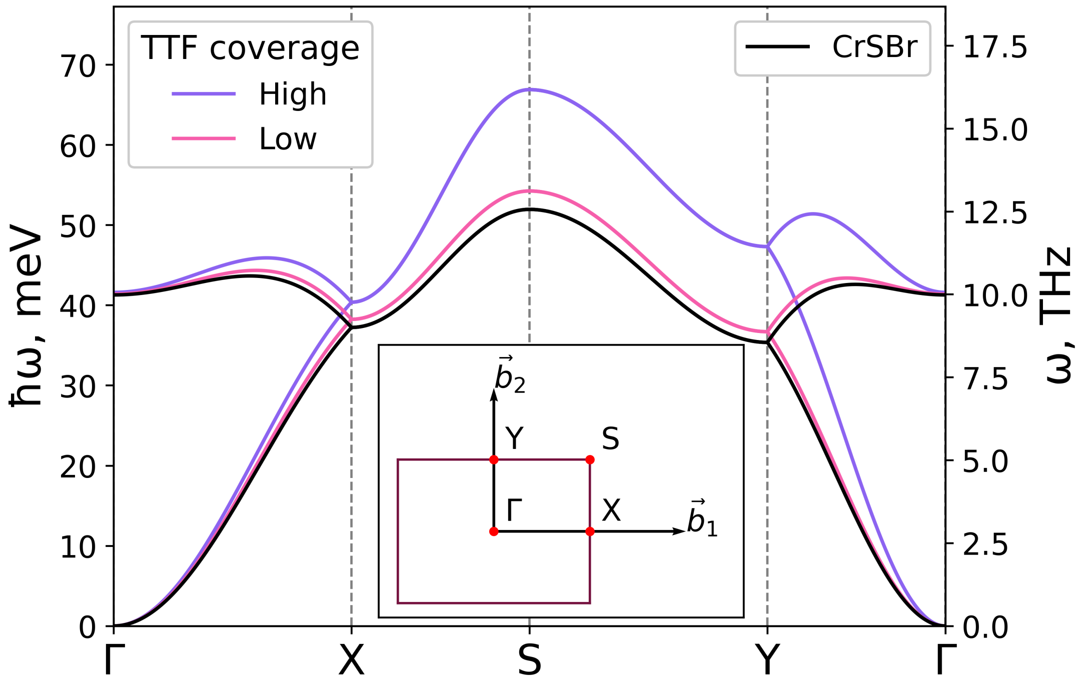

Fig. S28. Magnon dispersion for pristine CrSBr and different coverages of TTF.

4.2 Group velocities

Group velocities of magnon propagation are defined as a derivative of the frequency with respect to the chosen direction of ***k***. Due to the orthorhombic symmetry of the lattice, we focus our attention on the dependency of the group velocity ($\boldsymbol{v_x}$) in the direction of the first lattice vector (*a*) with the ***k*** vector along Γ-X path and of the group velocity ($\boldsymbol{v_y}$) in the direction of the second lattice vector (*b*) with the ***k*** vector along Γ-Y path. The corresponding formulas are:

$$v_x(\boldsymbol{k}_{\Gamma-\mathrm{X}}) \;=\; v_x^{\Gamma-\mathrm{X}} = 4Sa\left(J_1 \sin(k_x a) \;\pm J_2 \sin\left(\frac{k_x a}{2}\right)\right)$$

$$v_y(\boldsymbol{k}_{\Gamma-\mathrm{Y}}) \;=\; v_y^{\Gamma-\mathrm{Y}} = 4Sb\left(J_3 \sin(k_y b) \;\pm J_2 \sin\left(\frac{k_y b}{2}\right)\right)$$

In Figs. S29, S30 the absolute values of the group velocities are presented for: molecules on top of the substrate; distortion + electron doping.

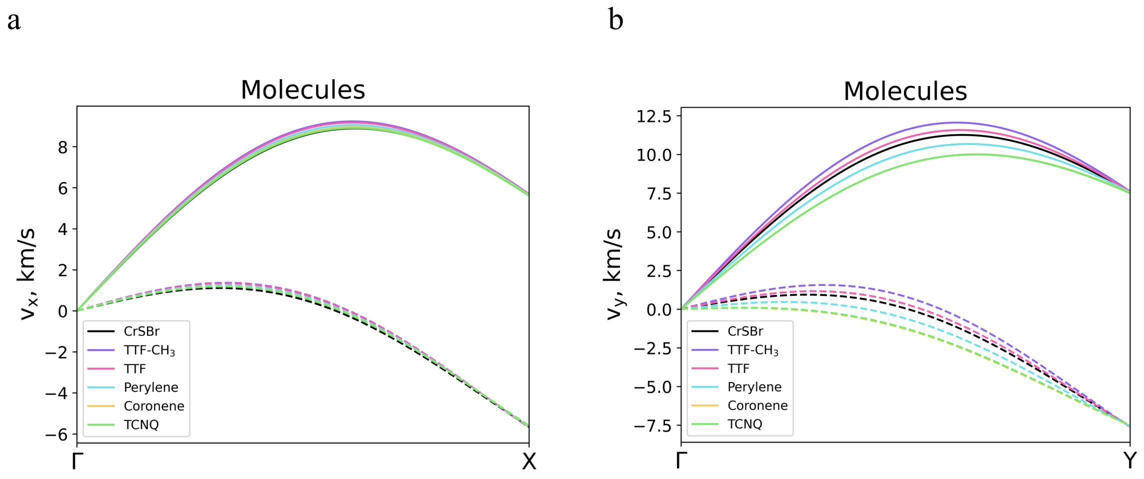

Fig. S29. Group velocities of the magnon propagation for the CrSBr substrate with the molecules deposited on top. (a) Group velocity $v_x^{\Gamma-X}$ (b) Group velocity $v_y^{\Gamma-Y}$. Solid lines for the acoustic branch, dashed lines for the optical branch.

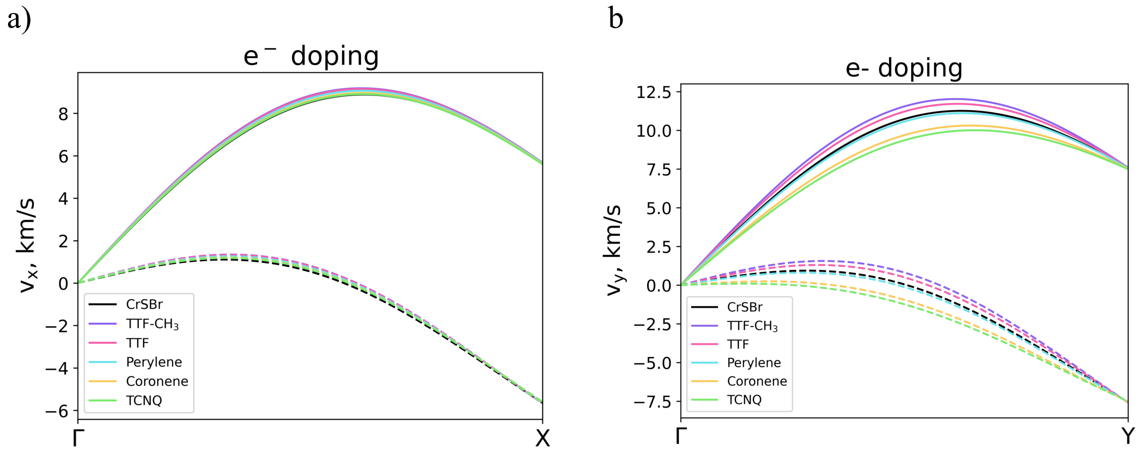

Fig. S30. Group velocities of the magnon propagation for the CrSBr substrate with the distortion and electron doping, corresponding to each molecule. (a) Group velocity $v_x^{\Gamma-X}$ (b) Group velocity $v_y^{\Gamma-Y}$. Solid lines for the acoustic branch, dashed lines for the optical branch.

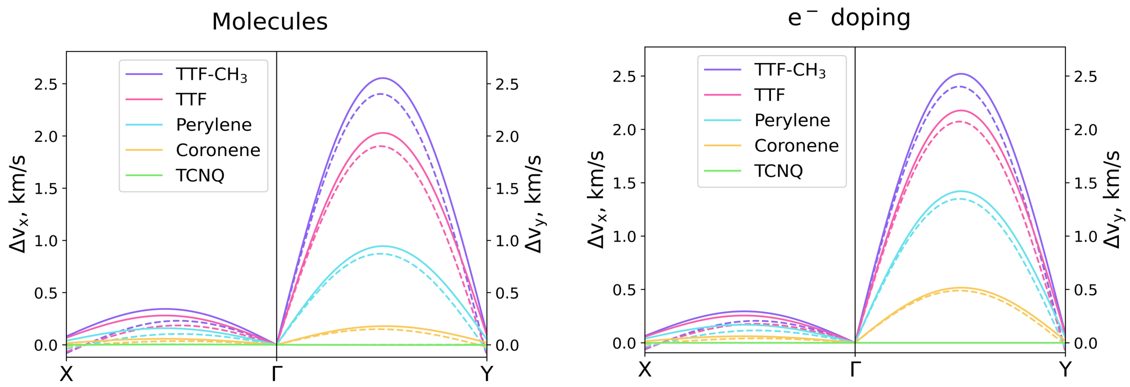

Fig. S31. Effect of the (a) molecules and (b) electron doping on the group velocities with respect to the corresponding distortion.

Relative change in group velocity in the Fig. S30 is defined as:

$$\Delta v_\alpha(\boldsymbol{k}) \; = \; v_\alpha(\boldsymbol{k}) \; - v_\alpha^{\mathrm{CrSBr}}(\boldsymbol{k})$$

where α stands for *x* or *y*. ***k***-dependent, relative (to the value for pristine CrSBr) group velocity is defined as:

$$v_\alpha^{norm}(\boldsymbol{k}) \; = \; \frac{v_\alpha(\boldsymbol{k})}{v_\alpha(\boldsymbol{k})^{CrSBr}}$$

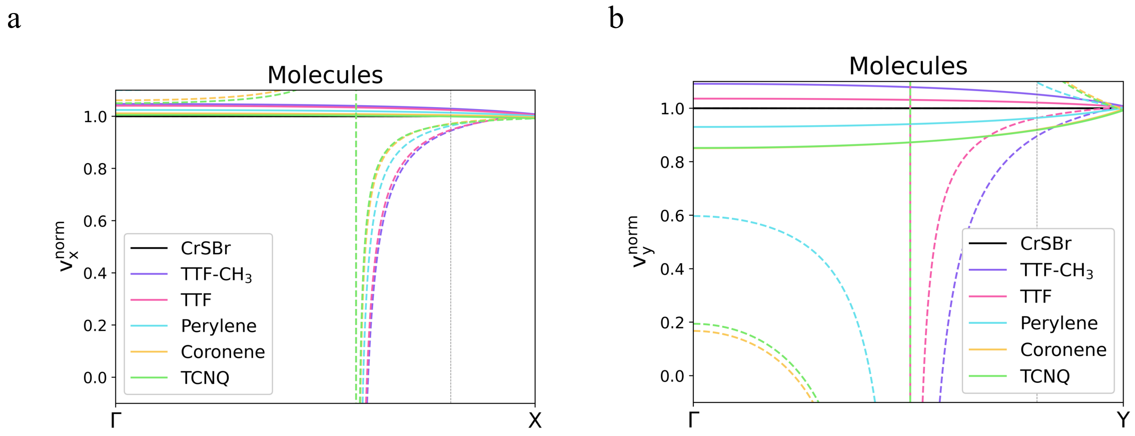

Fig. S32. Relative group velocity of the magnon propagation in the CrSBr substrate with the molecules deposited on top. (a) v_x^{norm} (b) v_y^{norm}. Solid lines for the acoustic branch, dashed lines for the optical branch.

The relative group velocity for the acoustic branch, for the low-energy magnons does not depends on the ***k*** vector significantly. Therefore, in the **Fig. 4**d of the main text we plot the mean value for the arbitrary chosen range of 80% of the corresponding k-path (Γ-X or Γ-Y). Grey dashed lines in the Fig. S32 indicate this range. In the **Table S10** the values and the linear fit (*y = ax + b*) from the Fig. 4d of the main text are presented.

Table S10. Data and fit parameters of the relative velocity vs induced charge transfer.

	C.T.	Δv_x^{norm}	Δv_y^{norm}
TTF-CH_3	0.800	0.9988	0.9988
TTF	0.692	0.9925	0.9541
Perylene	0.452	0.9785	0.8691
Coronene	0.164	0.9672	0.8049
TCNQ	-0.027	0.9617	0.8052
Fit			
y = ax+b			
a		0.045	0.243
b		0.961	0.786

5. Bibliography

1 Rybakov A. RAD-tools https://rad-tools.org (version 0.8.9).
2 T. Holstein and H. Primakoff, *Phys. Rev.*, 1940, **58**, 1098–1113.
3 A. Scheie, M. Ziebel, D. G. Chica, Y. J. Bae, X. Wang, A. I. Kolesnikov, X. Zhu and X. Roy, *Adv. Sci.*, 2022, **9**, 2202467.
